# Hydrodynamical pathways in the phase change of real fluids

**DOI:** 10.1038/s42005-026-02639-y

**Published:** 2026-04-27

**Authors:** Mirko Gallo, Filippo Occhioni, Riccardo Daniele, Carlo Massimo Casciola

**Affiliations:** Dipartimento di Ingegneria Meccanica e Aerospaziale, Sapienza Università di Roma, Rome, Italy

**Keywords:** Fluid dynamics, Phase transitions and critical phenomena

## Abstract

Identifying the incipient conditions of liquid-vapour transformation, the number of bubbles formed, their spatiotemporal scales, and the role of inertia remains a major challenge, reflecting how elusive the early stages of phase change are. Here, we present a theoretical framework that combines large deviation theory, multiphase fluctuating hydrodynamics and real fluid thermodynamics to compute the most probable nucleation pathways in metastable liquids. We identify the optimal trajectories connecting metastable and stable states and determine the full spatiotemporal structure of the nucleation process. Our results reveal that nucleation is not solely governed by thermodynamic forces, but is also shaped by hydrodynamic phenomena such as wave propagation and inertial effects. The approach predicts boiling thresholds for water, nitrogen, and helium, in agreement with experiments. It provides a unified, predictive description of phase-change kinetics linking microscopic fluctuations to macroscopic hydrodynamic observables, opening routes to prediction and control of phase change.

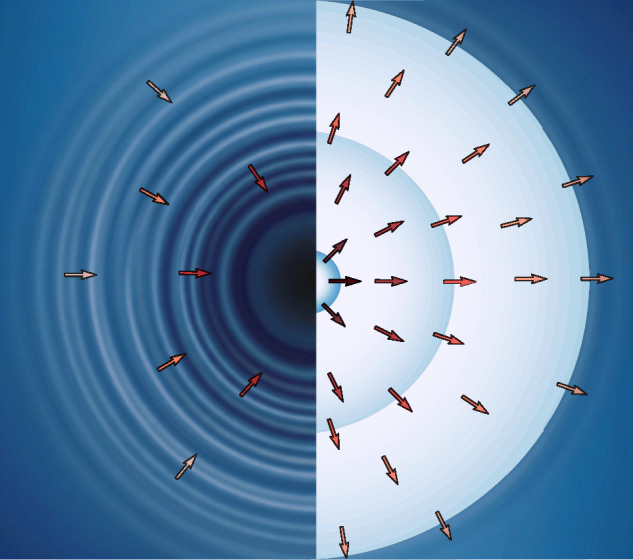

## Abstract (web summary)

Incipient stages of liquid-vapour phase change remain poorly understood because nucleation is an intrinsically rare, multiscale process. Here, the authors develop a theoretical framework that integrates large deviation theory, multiphase fluctuating hydrodynamics, and fluid thermodynamics to compute the most probable nucleation pathways in fluids, and they validate it by accurately predicting boiling thresholds for water, nitrogen, and helium.

## Introduction

A plethora of strategic next-generation technologies involve phase change. Thin-film deposition procedures^1^, atomic layer nucleation engineering in semiconductor technologies^2^, crystal nucleation in pharmaceutics^3^, and lithium deposition in batteries^4^ are only a few examples. Focusing on fluids, the continued miniaturisation of micro- and nanoscale systems has intensified the demand for effective thermal management^5^. Two-phase cooling is among the most promising strategies, which leverages latent heat through boiling and condensation^6^. Bubble nucleation is also exploited for energy conversion in electrochemical reactors^7^. However, the deployment of such technologies is fundamentally limited by our lack of predictive understanding of nucleation—the rare, stochastic process that initiates phase transitions under metastable conditions. For example, we still lack definitive answers to basic questions about the timing, progression, and measurable footprints of boiling/cavitation. Providing a holistic approach to answering these questions is the topic of the present work.

Nucleation arises from molecular fluctuations that overcome a free-energy barrier, triggering the formation of a new phase. These events are intrinsically rare, multiscale, and unfold over a wide range of time and length scales-from atomistic dynamics to macroscopic hydrodynamics-making them exceptionally difficult to model or observe directly^8^. To have an idea, the typical fluctuation time scale of molecules is on the order of 10^−13 ^s, while vapour bubble formation may require hours to occur^9–11^. Concerning space, critical bubble nuclei are nanometric objects, while bubble dynamics involves macroscopic scales.

For these reasons, there is a noticeable trend in computational fluid dynamics to embrace multiscale simulations, aiming to bridge molecular-scale phenomena with continuum-level fluid behaviour. This effort is powered by recent advances in parallelisation, GPU computing, and supercomputing, which now allow in-silico studies of fluids with billions of degrees of freedom. Yet, within this formidable scientific progress, one key limitation remains: the initial onset of phase change (nucleation) is still elusive. Its rarity and spatiotemporal complexity render it the missing link between atomistic mechanics and continuum multiphase models.

Existing continuum approaches rely on empirical closures and ad hoc assumptions^12^, while atomistic simulations are computationally infeasible at application-relevant scales^13^. Experimental investigations, too, are constrained by spatiotemporal resolution and system control^14^. As a result, the modelling of phase change remains largely phenomenological, impeding predictive design and optimisation in thermally demanding technologies.

Recent advances in mesoscale fluctuating hydrodynamics (FH) simulations have successfully addressed many of the challenges associated with the inherently multiscale nature of phase transition processes^15–18^. These techniques now enable the simulation of bubble formation from the very onset of nucleation up to the subsequent hydrodynamic evolution, bridging length scales from nanometric critical nuclei to micrometric bubble dynamics. However, despite these significant improvements, current FH approaches remain limited to temporal scales on the order of microseconds. As such, they are not yet capable, through brute-force simulations, of capturing bubble formation over the much longer timescales often observed under low metastability, which may be relevant for several technological applications, e.g. in two-phase heat management where bubble formation occurs on the order of seconds.

In this work, by combining diffuse interface hydrodynamics, real fluids’ equation of state, fluctuating hydrodynamics and large deviation theory, we close this gap by proposing a theoretical and computational framework that accurately describes nucleation as a rare, out-of-equilibrium, multiscale process. The proposed theory identifies the most likely transition path (MLP) for vapour bubble nucleation within the framework of hydrodynamic equations. When projected onto the reduced variables-the bubble radius *R* and the average density inside the bubble *ρ*_av_ the theoretical MLP is corroborated by brute-force fluctuating hydrodynamics simulations, unveiling a complex nucleation pathway reminiscent of modern theories of crystal formation^19^. Nucleation initiates at large radii and slightly rarefied liquid, progressing through a simultaneous decrease in radius and density-markedly contrasting with classical nucleation theory. Furthermore, the MLP uncovers a series of unexpected kinetic effects, highlighting in particular the role of underdamped dynamics in the formation of vapour bubbles. In contrast to theories based on overdamped dynamics, where inertial and compressibility effects are neglected, the approach proposed here captures the full interplay between density, momentum, and pressure fluctuations, showing that nucleation proceeds through the amplification of long-wavelength acoustic modes. These underdamped features, absent in purely diffusive models, highlight the critical role of hydrodynamics in driving rare nucleation events. After identifying the MLP, we estimate the boiling temperature by evaluating the nucleation rate across different real fluids and comparing our predictions with experimental data, finding excellent agreement. This work provides a comprehensive framework for understanding the nucleation process in real metastable liquids and the ensuing hydrodynamics, offering insights into the physics of phase transitions under realistic conditions.

## Results

The objective is to identify nucleation pathways and determine whether dynamical effects, such as acoustic or kinetic phenomena that were previously ignored, may be significant for phase transitions. Unlike overdamped methods or free energy-based techniques, the present approach preserves the intrinsic time scales of the transition process, enabling us to elucidate the mechanism driving the dynamics of nucleation.

The simulations described below were performed for water with *ρ* = 684.2 kg m^−3^ and *θ* = 302 °C, using the IAPWS-95^20^ equation of state (EoS) modified as in ref. ^11^. The theoretical aspects and the details of the calculations are reported in §  Methods.

### Nucleation mechanism and hydrodynamics

**Fig. 1:**
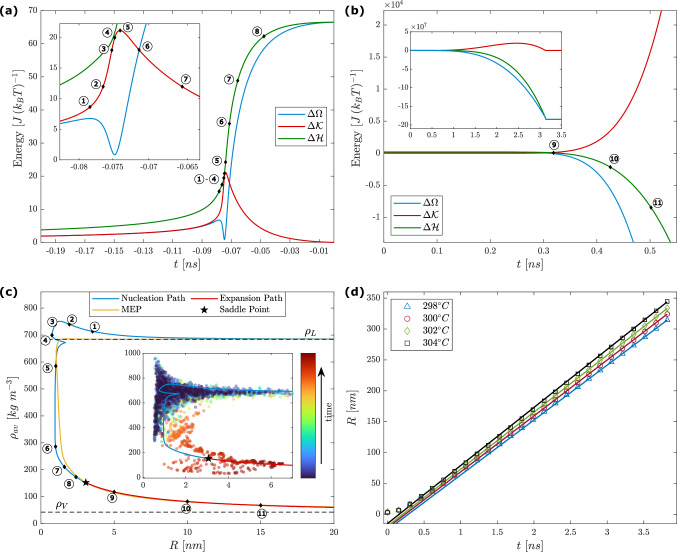
Energy landscape and bubble evolution along nucleation and expansion pathways. **a** Energy profiles versus time during the nucleation stage. The green line represents the total free energy $\Delta {{\mathcal{H}}}$, the red line the kinetic energy $\Delta {{\mathcal{K}}}$, and Δ*Ω* the grand potential. Numbers denote different time instants; for clarity, they are shown only along the total free energy curve. Inset: close-up of the region with the largest variations in kinetic energy and grand potential. Time instants are indicated only along the kinetic energy (red) curve for clarity. **b** As in (**a**), showing the total, kinetic and grand potential energies, now. The inset depicts larger times. **c** Nucleation path projected onto the reduced variables (*ρ*_av_, *R*). The blue and red curves, together with the saddle point, define the most likely path (MLP). The yellow curve represents the minimum energy path (MEP). *ρ*_*L*_ = 684.2 kg m^−3^ and *ρ*_*V*_ = 42.6 kg m^−3^. Inset: Comparison between MLP and CLLNS (fluctuating hydrodynamics) simulations. The coloured circles represent CLLNS stochastic realisation on the entire transition pathways from blue (short time) to red (long time). Consistently, blue circles relate to the nucleation stage, while the red symbols refer to the bubble expansion. Blue and red lines indicate the two corresponding pathways of the MLP. **d** Bubble radius as a function of time during the expansion stage. Different colours refer to different temperatures. Solid lines represent the Rayleigh-Plesset prediction, and symbols refer to the MLP.

Panels (a, b) of Fig. 1 show the total free energy $\Delta {{\mathcal{H}}}(t)$ as a function of time *t*, represented by the green curve. *t* = 0 corresponds to the critical state, while *t* → ∓ *∞* are the metastable (**X**_1_, liquid) and stable (**X**_2_, vapour) states, respectively. The kinetic energy $\Delta {{\mathcal{K}}}(t)$ is shown as the red curve and the Grand Potential Δ*Ω* as the light blue curve. All energetic contributions have been shifted by subtracting the energy (${{{\mathcal{H}}}}_{-\infty }$) of the metastable state **X**_1_, namely $\Delta {{\mathcal{H}}}={{\mathcal{H}}}[\rho ({{\bf{x}}},t),{{\bf{v}}}({{\bf{x}}},t)]-{{{\mathcal{H}}}}_{-\infty }$.

Focusing on panel (a)—the nucleation phase—the circled numbers identify different instants. During this phase, $\Delta {{\mathcal{H}}}(t)$ is monotonically increasing, as expected from considerations reported in Supplementary Note 8, on a temporal scale of order 20 ps (see inset). The kinetic energy $\Delta {{\mathcal{K}}}(t)$ is not monotonic, starting from zero (metastable quiescent liquid) and coming back to rest at the transition point. Unlike overdamped dynamics, whereΔ*Ω* is always increasing and $\Delta {{\mathcal{K}}}=0$, here the coupling between Landau free energy and kinetic energy is apparent.

Panel (b) of Fig. 1 refers to the supercritical bubble expansion state when the system has crossed the separatrix and evolves toward the eventual vapour state. We observe that this branch of the MLP corresponds to the deterministic relaxation described by the CNS system (zero noise limit). The colours denote the same physical quantities as in panel (a). Here, as expected, $\Delta {{\mathcal{H}}}$ is always decreasing, and the kinetic energy steadily increases, though less than the decrease of the grand potential. The inset shows the total free energy and its individual contributions at longer times. The kinetic energy increases to a maximum and subsequently decays to zero as the system fully transforms into vapour. In contrast, the total energy stabilises at a lower value corresponding to the (stable) vapour state, compared to the initial metastable liquid.

Panel (c) of Fig. 1 depicts the MLP projected onto two reduced variables in the (*R*, *ρ*_av_) space, where

$$R(t)=\frac{{\int }_{0}^{\infty }r\,{\left({\partial }_{r}\rho (r,t)\right)}^{2}{{\rm{d}}}r}{{\int }_{0}^{\infty }\,{\left({\partial }_{r}\rho (r,t)\right)}^{2}{{\rm{d}}}r}\,,\,\,\,\,{\rho }_{{{\rm{av}}}}(t)=\frac{3}{{R}^{3}}{\int }_{0}^{R}{r}^{2}\rho (r,t){{\rm{d}}}r.$$

The MLP consists of two curves in the (*R*, *ρ*_av_) space: the blue curve represents the nucleation phase, while the red curve corresponds to the expansion phase, with the saddle point indicated by a black star. Contrary to classical nucleation theory (CNT), which assumes that the bubble radius or volume alone suffices to describe the nucleation process, the MLP reveals a considerably more complex phenomenology. As it will be explained in detail later, nucleation begins at huge scales (putatively infinite) with the bubble average density exhibiting an initial increase, followed by a nontrivial decrease that culminates in the formation of the critical nucleus. The comparison with the MEP^21^, shown in dark yellow, indicates that the phenomenology is fairly similar, but in the MLP, there is an observed increase in the nucleus’s average density during the early nucleation phase. This effect is linked to inertial effects (underdamped dynamics), where waves generated at large scales propagate inward, compressing the fluid inside the nucleus. The inset shows a brute-force simulation performed with the CLLNS equations; the MLP lies within the cloud of points representing individual realisations of the nucleation process, with circles indicating the system state at a given time and different colours denoting different times, i.e. blue tones for precritical states and red tones for postcritical ones.

After nucleation occurs, the dynamics can be described by Rayleigh-Plesset (RP) dynamics. Panel (d) of Fig. 1 reports the bubble radius as a function of time and its comparison with the RP dynamics, see Supplementary Note 7 for details. As observed, provided that the RP dynamics are initialised at a sufficiently large scale—*t*≃1 ns after the nucleation event for IAWPS water corresponding to *R* ≃ 50 nm— the subsequent phases show good agreement. For a highly metastable liquid, such as water at approximately  ≃ 300°C, the expansion velocities become significant, ≃ 100 m s^−1^, as observed in explosive boiling.

**Fig. 2:**
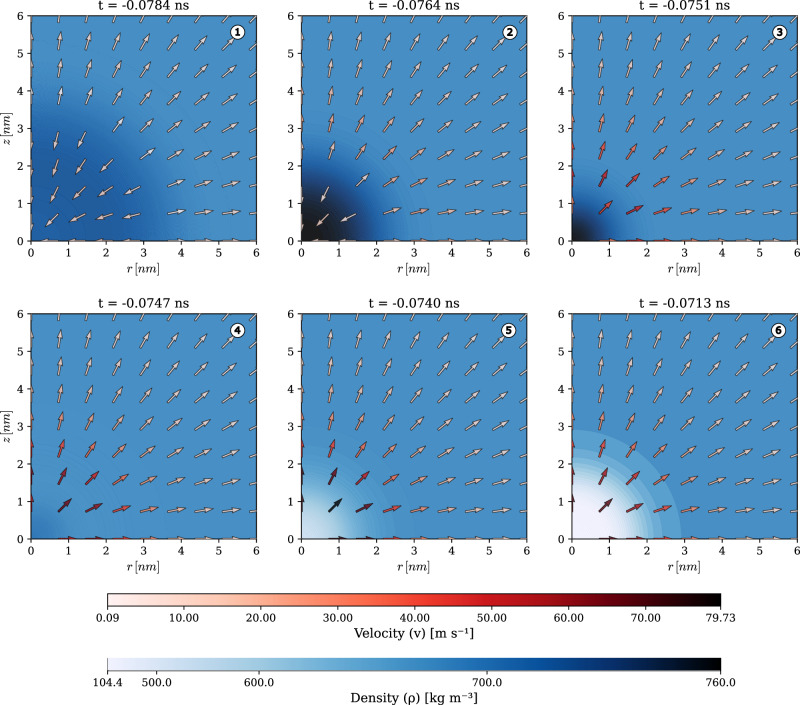
Density and velocity fields in the nucleation pathway. Snapshots of density contour plots and velocity vector fields in the precritical state at selected times, indicated by circled numbers corresponding to those in Fig. 1a. The fields are shown as a two-dimensional cut of a three-dimensional spherically symmetric field, corresponding to a quarter-plane in the meridional *r*-*z* plane. Density is shown in blue, with lighter (darker) shades corresponding to lower (higher) values; the colour bar is reported on a logarithmic scale for clarity. Velocity vectors are shown in red, with lighter (darker) shades indicating lower (higher) magnitudes.

**Fig. 3:**
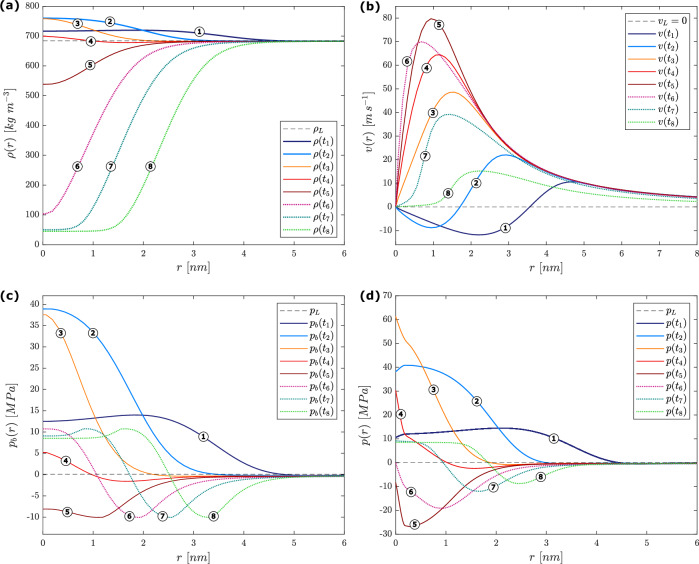
Radial profiles of density, velocity and pressure along the nucleation pathway. **a** Density profiles along the MLP during the nucleation stage. **b** Velocity profiles along the MLP during the nucleation stage. **c** Bulk pressure profiles along the MLP during the nucleation stage. **d** Generalised pressure profiles along the MLP during the nucleation stage. Circled numbers indicate different time instants (see Fig. 1a) and are also reported in the corresponding legend. Dotted lines correspond to the phase in which the kinetic energy decreases, while solid lines refer to the phase in which it increases. Black dashed lines denote the metastable fields, namely the liquid density (*ρ*_L_), velocity (*v*_L_), bulk/generalised pressure (*p*_L_). *r* corresponds to the radial coordinate.

To provide a pictorial overview of the process, Fig. 2 presents the density and velocity vector fields, illustrating the qualitative features of nucleation, before we discuss in detail the individual fields composing the MLP shown in Fig. 3.

Panel (a) of Fig. 3 shows the density fields along the MLP. The numbers inside the circles correspond to different time instants and are marked consistently with panel (a) of Fig. 1, which describes the energy variation over time. In the initial phase, a compression of the liquid is observed, resulting from the compression wave generated at large distances (see the next figure for details) towards the centre of the forming nucleus. As the wave propagates, the liquid gets compressed (kinetic energy grows), with profiles marked by numbers 1–4, and then relaxes, initiating the classical bubble formation phase, with profiles marked by numbers 5–8.

This acoustic effect is not captured by the overdamped dynamics (MEP), where density profiles monotonically increase with distance from the nucleus centre; see Supplementary Note 5.

Panel (b) of Fig. 3 reports the velocity fields along the MLP. Consistent with the density observations, nucleation starts with a wave propagating from large distances toward the centre. The velocity significantly increases during this process, starting from an alternating-sign condition (profiles 1 and 2; see the subsequent figure for its structure at large *r*), becoming purely compressive up to a maximum of about 80 m s^−1^ (profiles 3–5), and then begins to decelerate (profiles 5–8), to vanish at the saddle point.

Panels (c) and (d) of Fig. 3 show the pressure fields along the MLP. Panel (c) presents the bulk pressure *p*_b_ = *ρ*∂_*ρ*_*f*_b_ − *f*_b_, while panel (d) displays the generalised pressure $p={p}_{{{\rm{b}}}}-\lambda /2{({\partial }_{r}\rho )}^{2}-\lambda \rho /{r}^{2}{\partial }_{r}({r}^{2}{\partial }_{r}\rho )$. In both panels, the compression wave appears to drive the vapour formation process: the liquid strongly compresses (profiles 1–4) and then starts to rarefy (profiles 5–8), reaching negative pressures on the order of tens of MPa. We stress that critical nuclei are comparable in size to the interface thickness, implying that density gradients make the generalised pressure differ from the bulk pressure.

**Fig. 4:**
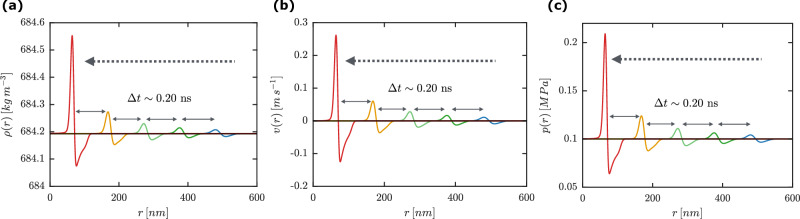
Far-field density, velocity ad pressure radial profiles at different times. **a** Density profiles for large *r*. **b** Velocity profiles for large *r*. **c** Generalised pressure profiles for large *r*. Different colours correspond to different time instants; the arrow indicates the direction of propagation. The black horizontal solid lines denote the metastable fields, namely the liquid density (*ρ*_L_), velocity (*v*_L_), bulk/generalised pressure (*p*_L_). *r* corresponds to the radial coordinate.

Panels (a–c) of Fig. 4 show the density, velocity, and pressure fields, respectively, at large distances from the nucleus centre. These fields are the precursors of the nucleation process, i.e., the precursors (*t* → −*∞*) of the most probable trajectory towards vapour nucleation (it may be worth stressing that, in the present context, trajectories even slightly different from the instanton are expected to have exponentially vanishing probability). The different colours correspond to different times, and the arrows indicate the direction of wave propagation. The time between two different peaks is also reported, i.e. Δ*t* ~ 0.20 ns. For *t* → −*∞*, waves of tiny amplitude are observed at increasingly large distances from the nucleus centre. As *t* increases, the amplitude of these waves gradually intensifies while they focus toward the centre, triggering the nucleation phase. All such waves have a wavelength of approximately 90 nm and a propagation speed of about 450 m s^−1^, which corresponds to the isothermal sound speed ${c}_{\theta }=\sqrt{{\partial }_{\rho }{p}_{{{\rm{b}}}}{| }_{\theta }}$ with *ρ* = 684.2 kg m^−3^ and *θ* = 302 °C. The typical frequency of these waves is in the gigahertz range.

**Fig. 5:**
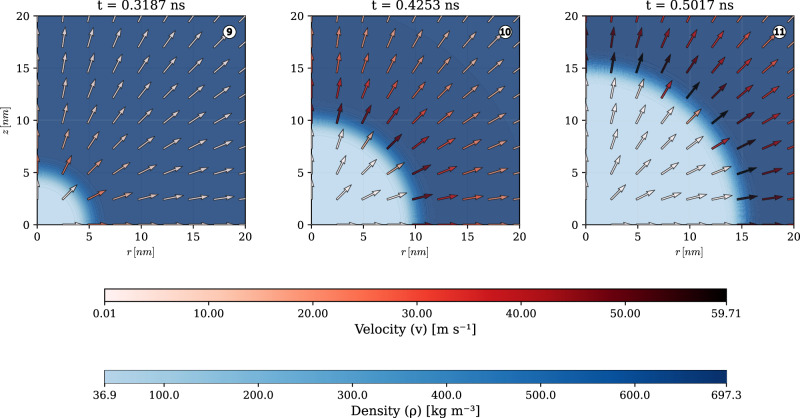
Density and velocity fields during bubble expansion. Snapshots of density contour plots and velocity vector fields in the postcritical state at selected times, indicated by circled numbers corresponding to those in Fig. 1b. The fields are shown as a two-dimensional cut of a three-dimensional spherically symmetric field, corresponding to a quarter-plane in the meridional *r*–*z* plane. Density is shown in blue, with lighter (darker) shades corresponding to lower (higher) values. Velocity vectors are shown in red, with lighter (darker) shades indicating lower (higher) magnitudes.

**Fig. 6:**
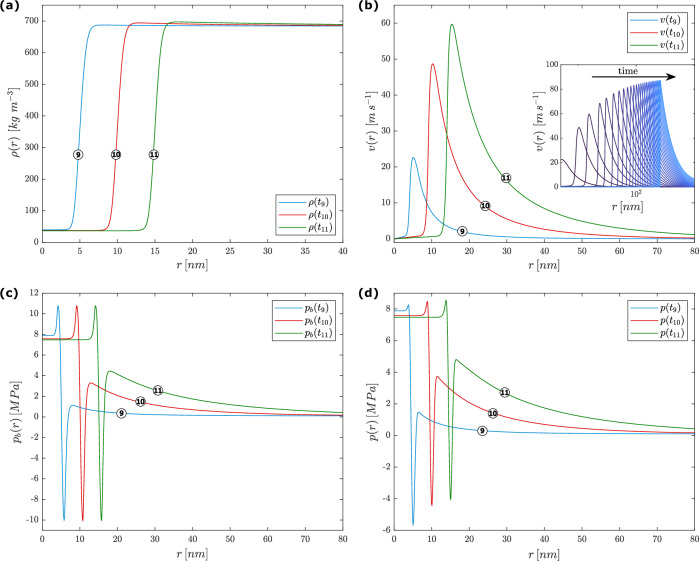
Radial profiles of density, velocity, and pressure along the expansion pathway. **a** Density profiles along the MLP during the expansion stage. **b** Velocity profiles along the MLP during the expansion stage. Inset: velocity fields at large radii, different curves correspond to different times. **c** Bulk pressure profiles along the MLP during the expansion stage. **d** Generalised pressure profiles along the MLP during the expansion stage. Circled numbers indicate different time instants (see Fig. 1b) and are also reported in the corresponding legend. *r* corresponds to the radial coordinate.

Figure 5 provides a pictorial overview of the fields, before we analyse in detail the postcritical evolution shown in Fig. 6.

Panel (a) of Fig. 6 shows the density fields along the MLP, during the expansion phase of the supercritical bubble. The numbers inside the circles correspond to different times consistently with panel (a) of Fig. 1. The density profiles gradually shift towards larger radii as time progresses, as shown in profiles 9–11. Unlike the overdamped dynamics, they show a slight compression of the liquid due to the liquid’s inertia and the compressibility of the liquid phase.

Panel (b) of Fig. 6 reports the velocity fields, whose peak grows over time during the initial phases. This panel captures the more elusive part of the process, specifically the phases that immediately follow critical bubble formation. The inset focuses on the velocity fields at large radii for different time instants. Consistently with panel (d) of Fig. 6, it shows that at large radii the velocity plateaus to the limiting value of the RP dynamics, ${\dot R}=\sqrt{2\Delta p/(3{\rho }_{{{\rm{L}}}})}$,^22^, with Δ*p* the pressure difference between the inside and outside of the bubble, and *ρ*_L_ the density of the metastable liquid.

Panels (c) and (d) of Fig. 6 report the pressure fields along the MLP. Panel (c) shows the bulk pressure, while panel (d) shows the generalised pressure. In both panels, two characteristic positive and negative pressure peaks associated with the presence of the interface can be distinguished. These peaks are reduced in amplitude when considering the generalised pressure. The compression phase induced by the expansion discussed in the density profiles is also present in the pressure fields, and it attenuates as *r* increases.

### Boiling temperatures and comparison with experiments

In this section, we aim to identify the boiling temperatures (superheat limit) of three different fluids and compare them with available experimental data^23^. In line with experimental approaches, we define the operating conditions for the phase transition in terms of pressure and temperature as those for which there is a probability of 1/2 of observing at least one bubble nucleation within a time window $\bar{T}$ and a volume *V*^9^. Assuming that nucleation is a Poisson process, as recently shown by large-scale FH simulations^24^, the probability of boiling is given by ${p}_{n\ge 1}=1-\exp (-JV\bar{T})$, where *J*(*p*, *θ*) is the nucleation rate. The boiling temperature is defined such that $J=ln2/(V\bar{T})={J}_{{{\rm{crit}}}}$. For *J*, we used the expression derived in §Methods.

1 $$J(p,\theta )=0.245\frac{{\sigma }^{11/2}{c}_{\theta }^{4}}{{({k}_{{{\rm{B}}}}\theta )}^{3/2}{(\Delta p)}^{3}{\rho }_{{{\rm{L}}}}^{2}{\lambda }^{2}{\eta }_{1}}\exp \left(-\frac{\Delta {\Omega }^{\star }}{{k}_{{{\rm{B}}}}\theta }\right)\,,$$

where Δ*p* = *p*_V_ − *p*_L_ is the pressure difference between the centre of the bubble and the metastable liquid, *σ* is the surface tension of the critical nucleus, and *c*_*θ*_ is the isothermal speed of sound. Curvature dependence of surface tension (Tolman-like correction) is crucial for nucleation. Our model captures it because the DFT barrier automatically includes the curvature correction^11,25^; taking a constant planar *σ* only weakly affects the prefactor but is decisive for the barrier that enters the exponential (in CNT, Δ*Ω*^⋆^ ~ *σ*^3^). In experiments, a threshold nucleation rate *J*_crit_ is typically defined, which can vary from 10^6^ to 10^18^ critical bubbles per cubic metre per second^26^, corresponding approximately to a single nucleation event per second in a volume ranging from cm^3^ to μm^3^. However, as will be shown, the boiling temperature is not strongly sensitive to the exact value of *J*_crit_.

**Fig. 7:**
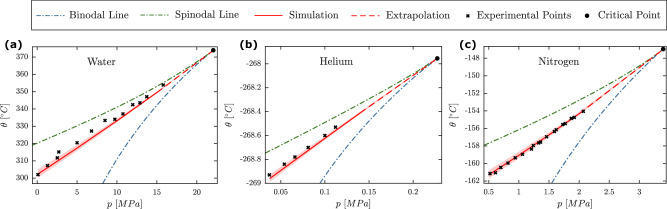
Thermodynamic phase diagram and homogeneous nucleation limit for water, helium, and nitrogen. Temperature (*θ*)--pressure (*p*) phase diagrams for **a** Water. **b**Helium. **c** Nitrogen. The binodal (blue dash-dotted line), spinodal (green dash-dotted line), and experimental data (black crosses) are shown together with the boiling curve predicted by our framework (solid red line). The light red shaded region represents the uncertainty associated with the choice of the critical nucleation rate (*J*_crit_), here taken in the range 10^6^–10^18^, and thus provides an estimate of the error on the predicted boiling curve.

Panels (a–c) of Fig. 7 show, in the (*p*, *θ*) plane, the binodal (blue), spinodal (green), and boiling (red) curves for water, helium, and nitrogen, respectively. The boiling temperature is estimated by fixing *J*_crit_ = 10^12^ m^−3^ s^−1^, and the surrounding light red bands indicate the variation in boiling temperature corresponding to lower and upper bounds of *J*_crit_ = 10^6^ m^−3^ s^−1^ and 10^18^ m^−3^ s^−1^, respectively. At a given pressure *p*_0_, the boiling temperature *T*_boil_ is defined as the solution of the equation *J*(*p*_0_, *T*_boil_) = *J*_crit_.

Experimental results, shown as black symbols, are taken from the review^23^, and the comparison is favourable for all three fluids considered.

## Discussion

This work presents a hydrodynamic-level description of nucleation, providing a physical understanding of how phase change initiates under metastable conditions. Our results show that nucleation is triggered by long-wavelength acoustic modes that gradually amplify over time, eventually leading to the formation of a critical nucleus. The transition pathway (MLP) in the nucleation stage exhibits the same reversible component as classical hydrodynamics, while the dissipative component appears with an opposite sign. It is as if an ‘anti-dissipative’ mechanism were driving the system uphill across the free energy barrier. This behaviour indicates that nucleation in this regime is not a purely local fluctuation but rather a collective process governed by long-range hydrodynamic modes. In addition, our approach provides direct access to the characteristic time scales of all processes involved in nucleation, revealing the typical wavelengths (on the order of 90 nm) and frequencies (in the gigahertz range) that drive the onset of the transition. This yields, at negligible computational cost (standard laptop), a comprehensive hydrodynamic view of phase change in real fluids, described using refined equations of state that faithfully capture the fluid’s thermodynamics. Beyond this, once the nucleation probability is quantified, the nucleation rate can be estimated, allowing us to infer the typical boiling temperatures of real fluids, with astonishing agreement with experimental data.

From a fundamental point of view, these findings open new perspectives for modelling phase transitions across scales, bridging microscopic fluctuation dynamics with continuum hydrodynamics. They call for a rethinking of how nucleation is treated in multiphase fluid models, where it is often reduced to a local and quasi-static criterion. It should, however, be emphasised that the small-*ϵ* approximation underlying the present framework corresponds to an asymptotic regime and is therefore delicate. In realistic systems, the effective value of *ϵ* associated with thermal fluctuations may not be strictly small. In the present context, *ϵ* should be compared with the typical magnitude of the action *S*_*T*_[**X**], which in nucleation problems can reasonably be expected to scale with the height of the free-energy barrier Δ*Ω*^⋆^. Since the path probability scales as ${{{\bf{P}}}}_{{{\rm{pc}}}} \sim \exp \left(-{S}_{T}[{{\bf{X}}}]/\epsilon \right)$ (see Eq. (18)), a natural dimensionless estimate of the noise strength is ϵ^*^ ~ *k*_B_*θ*/Δ*Ω*^⋆^. For the cases considered here, this yields ϵ^*^ ~ 10^−2^, which is fairly consistent with the small-noise hypothesis. The role of fluctuations beyond this asymptotic regime will be addressed in future work, where the nucleation pathways computed here can be exploited to design efficient rare-event sampling strategies.

We also note that the velocity equation (Eq. (3)) provides only an approximate description of the fluid dynamics. In the present formulation, interparticle interactions are accounted for through an equilibrium force density based on squared-gradient theory, which assumes local thermodynamic equilibrium at the instantaneous values of the hydrodynamic fields. This description is expected to be robust in the present setting, since the interfacial thickness is much larger than the range of the interaction potentials^27^. Dissipation is further described within a Newtonian framework, assuming small deviations from local equilibrium. Higher-order non-equilibrium corrections to the force density are neglected here.

From a technological perspective, the methodology provides a predictive and accurate estimate of the boiling (cavitation) limits of real liquids, provided that a reliable and thermodynamically consistent equation of state is available. Furthermore, it can be easily extended to condensation processes^28^. The nonclassical (*C*-shaped) nucleation pathways predicted by this approach (see Panel (c) of Fig. 1) have been observed in atomistic simulations of droplet nucleation^29^. Direct comparisons with molecular dynamics simulations remain a natural direction for future work.

As a final comment, the proposed technique is general and can be extended to a broad range of systems in which nucleation and its dynamics play a fundamental role. Potential applications include, for instance, crystal formation^19^, biomolecular condensates^30^, and active matter^31,32^. In all these contexts, capturing the interplay between fluctuations and hydrodynamic or collective dynamics is essential for a predictive understanding of phase transition pathways.

## Methods

### Capillary Landau-Lifshitz-Navier-Stokes equations (CLLNS)

Even at thermodynamic equilibrium, mesoscopic physical systems are significantly affected by thermal fluctuations, which must therefore be explicitly accounted for in any accurate theoretical description. Accordingly, the dynamics of a mesoscopic system are governed by the balance equations for mass and momentum, augmented by stochastic fluxes that capture the influence of thermal noise, here reported for isothermal systems at a constant temperature *θ*, see ref. ^15^ for details

2 $${\partial }_{t}\rho =-\nabla \cdot \left(\rho {{\bf{v}}}\right)\,,$$

3 $$\rho {\partial }_{t}{{\bf{v}}}=-\rho \left({{\bf{v}}}\cdot \nabla \right){{\bf{v}}}+\nabla \cdot {{{\bf{T}}}}_{{{\rm{visc}}}}+\nabla \cdot {{{\bf{T}}}}_{{{\rm{cap}}}}+\nabla \cdot \widetilde{{{\bf{T}}}},$$

where *ρ*(**x**, *t*) is the mass density, **v**(**x**, *t*) the velocity field.

4 $$\nabla \cdot {{{\bf{T}}}}_{{{\rm{cap}}}}=-\rho \nabla \left(\frac{\delta \Omega }{\delta \rho }\right),$$

with *Ω*[*ρ*, *θ*], the Landau (*grand potential*) free-energy

5 $$\begin{array}{l}\Omega [\rho ,\theta ]=F[\rho ,\theta ]-{\mu }_{{{\rm{eq}}}}{\int }_{{{\mathcal{D}}}}\rho {{\rm{d}}}V=\\ ={\int }_{{{\mathcal{D}}}}f(\rho ,\nabla \rho ,\theta )\,{{\rm{d}}}V-{\mu }_{{{\rm{eq}}}}{\int }_{{{\mathcal{D}}}}\rho {{\rm{d}}}V\,,\end{array}$$

where $F[\rho ,\theta ]={\int }_{{{\mathcal{D}}}}{f}_{{{\rm{b}}}}(\rho ,\theta )+\lambda /2| \nabla \rho {| }^{2}{{\rm{d}}}V$ is the Helmholtz free energy, *f*_b_(*ρ*, *θ*) the bulk free-energy density, which can accurately describe real fluids through an appropriate equation of state^33^, *μ*_eq_ the equilibrium chemical potential, and ${{\mathcal{D}}}$ the domain representing the fluid volume over which the free energy is evaluated.

The capillary coefficient *λ* is, in general, a function of temperature and determines the surface tension and the liquid/vapour interface thickness^11^. This thermodynamic framework corresponds to the squared gradient theory originally introduced by van der Waals^34,35^, which can be rigorously derived from the more general density functional theory (DFT)^36^ as a first-order gradient expansion. This approximation holds when density gradients are small, i.e. interface thickness much larger than the decay length of interaction potential (as is the case in all scenarios treated in this paper), but it has also been shown to agree well with experimental data even in situations with large gradients, such as water at room temperature^11^. The theory provides a generalised pressure, accounting for capillary effects *p* = *ρδF*/*δρ* − *f* = *p*_b_ − *λ*/2∣ ∇ *ρ*∣^2^ − *λρ*∇^2^*ρ*, with *p*_b_ = *ρ*∂*f*_b_/∂*ρ* − *f*_b_, the bulk pressure.

6 $$\nabla \cdot {{{\bf{T}}}}_{{{\rm{visc}}}}={\eta }_{1}{\nabla }^{2}{{\bf{v}}}+\left({\eta }_{2}+\frac{1}{3}{\eta }_{1}\right)\nabla \nabla \cdot {{\bf{v}}},$$

with *η*_1_ the shear and *η*_2_ the bulk viscosity coefficients. $\nabla \cdot \widetilde{{{\bf{T}}}}({{\bf{x}}},t)$ is a Gaussian vector process with zero mean and correlation

7 $$\begin{array}{l}\langle \nabla \cdot \widetilde{{{\bf{T}}}}({{\bf{x}}},t)\otimes \nabla \cdot {\widetilde{{{\bf{T}}}}}^{{\dagger} }({{\bf{y}}},q)\rangle =\\ =2{k}_{{{\rm{B}}}}\theta \left[{{\bf{I}}}{\eta }_{1}{\nabla }_{{{\bf{x}}}}^{2}+\left({\eta }_{2}+\frac{1}{3}{\eta }_{1}\right){\nabla }_{{{\bf{x}}}}\otimes {\nabla }_{{{\bf{x}}}}\right]\delta ({{\bf{x}}}-{{\bf{y}}})\delta (t-q),\end{array}$$

with **I** the identity matrix, ∇_**x**_ the gradient with respect to the variable **x** and k_B_ the Boltzmann constant.

### Transverse decomposition of CLLNS

The system of Eqs. (2) and (3) have an associated total free-energy ${{\mathcal{H}}}[\rho ,{{\bf{v}}}]$ (coarse-grained Hamiltonian) which corresponds to the sum of the Landau free energy *Ω*[*ρ*] and the kinetic energy ${{\mathcal{K}}}[\rho ,{{\bf{v}}}]={\int }_{{{\mathcal{D}}}}\frac{1}{2}\,\rho \,| {{\bf{v}}}{| }^{2}\,{{\rm{d}}}V$

8 $${{\mathcal{H}}}[\rho ,{{\bf{v}}}]=\Omega +{{\mathcal{K}}}={\int }_{{{\mathcal{D}}}}{f}_{{{\rm{b}}}}(\rho )+\frac{\lambda }{2}| \nabla \rho {| }^{2}-{\mu }_{{{\rm{eq}}}}\rho +\frac{1}{2}\rho | {{\bf{v}}}{| }^{2}\,{{\rm{d}}}V.$$

Let us define the vector ${{\bf{X}}}=\left(\rho ,{{\bf{v}}}\right)$, so that

9 $$\frac{\delta {{\mathcal{H}}}}{\delta {{\bf{X}}}}=\left(\mu +\frac{1}{2}| {{\bf{v}}}{| }^{2},\rho {{\bf{v}}}\right)\,,$$

with

$$\mu =\frac{\delta \Omega }{\delta \rho }=\frac{\partial {f}_{{{\rm{b}}}}}{\partial \rho }-\lambda {\nabla }^{2}\rho -{\mu }_{{{\rm{eq}}}},$$

the difference between the (generalised) chemical potential ∂_*ρ*_*f*_b_ − *λ*∇^2^*ρ* and the (assigned) equilibrium one *μ*_eq_. Therefore, Eqs. (2) and (3)) can be written as

10 $${\partial }_{t}{{\bf{X}}}=-{{\mathcal{T}}}\left[{{\bf{X}}}\right]-{{\mathcal{A}}}\,\frac{\delta {{\mathcal{H}}}}{\delta {{\bf{X}}}}+\sqrt{2\epsilon }\,\Sigma \,\xi$$

with

11 $${{\mathcal{A}}}=\left(\begin{array}{cc}0 & 0\\ 0 & \Gamma \end{array}\right)={{{\mathcal{A}}}}^{{\dagger} }\,,$$

a self-adjoint (Hermitian) operator and

12 $$\Gamma =-{\rho }^{-1}\left[{\eta }_{1}{{\bf{I}}}\,{\nabla }^{2}+\left({\eta }_{2}+\frac{1}{3}{\eta }_{1}\right)\nabla \otimes \nabla \cdot \right]{\rho }^{-1},$$

is the viscous contribution in CLLNS.

13 $${{\mathcal{T}}}\left[{{\bf{X}}}\right]=\left(\nabla \cdot \left(\rho {{\bf{v}}}\right),\left({{\bf{v}}}\cdot \nabla \right){{\bf{v}}}+\nabla \mu \right)$$

is the transport operator, and it satisfies the condition

$$\left({{\mathcal{T}}}\left[{{\bf{X}}}\right],\frac{\delta {{\mathcal{H}}}}{\delta {{\bf{X}}}}\right)={\int }_{{{\mathcal{D}}}}{\left({{\mathcal{T}}}\left[{{\bf{X}}}\right]\right)}^{T}\frac{\delta {{\mathcal{H}}}}{\delta {{\bf{X}}}}{{\rm{d}}}V=0,$$

which ensures that the total free energy of the system decreases (only) due to viscous dissipation

14 $$\frac{{{\rm{d}}}{{\mathcal{H}}}}{{{\rm{d}}}t}=-\left(\frac{\delta {{\mathcal{H}}}}{\delta {{\bf{X}}}},{{\mathcal{A}}}\frac{\delta {{\mathcal{H}}}}{\delta {{\bf{X}}}}\right).$$

The stochastic vector *ξ* is a zero-mean Gaussian process characterised by the correlation

15 $$\langle \xi ({{\bf{x}}},t)\otimes {\xi }^{{\dagger} }({{\bf{y}}},q)\rangle ={{\bf{I}}}\delta ({{\bf{x}}}-{{\bf{y}}})\delta (t-q)\,.$$

The fluctuation-dissipation balance imposes

16 $$\Sigma {\Sigma }^{{\dagger} }={{\mathcal{A}}}.$$

and *ϵ* = *k*_B_*θ*.

### Most likely path of nucleation (MLP)

In the following section, we aim to examine the connection between the nucleation process of a vapour bubble and the rare trajectories described by the stochastic CLLNS equations. The natural approach is based on large deviation theory which, since the seminal works in non-equilibrium statistical mechanics^37–39^, has been applied to various fields, including turbulence intermittency^40^, geophysical flows^41,42^, rogue waves^43^, film rupture^44^, and crystal nucleation^19^. Here, we use the theory to describe boiling in superheated fluids. Several attempts have been made to characterise fluctuation paths in stochastic systems, such as in the context of overdamped nucleation using dynamical density functional theory^45^, or within the Smoothed Particle Hydrodynamics (SPH) formulation of fluctuating hydrodynamics^46^, or in macroscopic fluctuation theory^47^. Similar approaches have also been employed to generalise Kramers’ theory in far-from-equilibrium settings^48^.

However, to the best of our knowledge, such fluctuation paths have not yet been computed within the CLLNS framework for a real fluid undergoing a phase change.

To this end, we consider the phase transition from a metastable liquid to a stable vapour as a trajectory **X**(**x**, *t*) connecting two states, **X**_1_ = (*ρ*_L_, 0) = **X**(**x**, *t* = − *T*) and **X**_2_ = (*ρ*_V_, 0) = **X**(**x**, *t* = *T*), over the specified time interval, 2*T*. The associated phase change probability ${{{\bf{P}}}}_{{{\rm{pc}}}}[{{\bf{X}}}]={{{\bf{P}}}}_{{{{\bf{X}}}}_{1}\to {{{\bf{X}}}}_{2}}$ is

17 $${{{\bf{P}}}}_{{{\rm{pc}}}}={{\bf{P}}}\left[{{\bf{X}}}({{\bf{x}}},T)={{{\bf{X}}}}_{2}| {{\bf{X}}}({{\bf{x}}},-T)={{{\bf{X}}}}_{1}\right].$$

In principle, there exists an infinite number of stochastic trajectories connecting the two end states. Each one can be interpreted as a possible realisation of a phase transition. The Freidlin-Wentzell theory assigns a probability to such trajectories in the limit of small *ϵ*^49^

18 $${{{\bf{P}}}}_{{{\rm{pc}}}} \sim \exp \left(-\frac{1}{2\epsilon }{S}_{T}[{{\bf{X}}}]\right),$$

by introducing the rate functional (action)

19 $${S}_{T}[{{\bf{X}}}]=\frac{1}{2}{\int }_{-T}^{T}{\left\Vert {\partial }_{t}{{\bf{X}}}+{{\mathcal{T}}}\left[{{\bf{X}}}\right]+{{\mathcal{A}}}\,\frac{\delta {{\mathcal{H}}}}{\delta {{\bf{X}}}}\right\Vert }_{{{{\mathcal{A}}}}^{+}}^{2}\,{{\rm{d}}}t,$$

where

$${\left\Vert {{\bf{Y}}}({{\bf{x}}},t)\right\Vert }_{{{{\mathcal{A}}}}^{+}}^{2}={\int }_{{{\mathcal{D}}}}{{\bf{Y}}}({{\bf{x}}},t){{{\mathcal{A}}}}^{+}{{{\bf{Y}}}}^{{\dagger} }({{\bf{x}}},t)\,{{\rm{d}}}V\,={\left(Y,Y\right)}_{{{{\mathcal{A}}}}^{+}},$$

and the Moore-Penrose pseudo-inverse (${{\mathcal{A}}}{{{\mathcal{A}}}}^{+}{{\mathcal{A}}}={{\mathcal{A}}}$) is

20 $${{{\mathcal{A}}}}^{+}=\left(\begin{array}{cc}0 & 0\\ 0 & {\Gamma }^{+}\end{array}\right).$$

The expression reported in Eq. (18) can be obtained by recognising the Gaussian probability distribution of the noise

21 $$P[\xi ]={\int }_{-T}^{T}\left(\xi ,\xi \right){{\rm{d}}}t\,,$$

and the relationship between the noise *ξ* and the trajectory **X** (*ξ* = *ξ*[**X**]). As shown in refs. ^45,50^, the dominant contribution to *P*[**X**] is the term of ${{\mathcal{O}}}({\epsilon }^{-1})$ (see Eq. (18)), while the subsequent terms, which are related to the Jacobian of the transformation *ξ* = *ξ*[**X**], scale as ${{\mathcal{O}}}({\epsilon }^{0})$. The probability of observing a given path decays exponentially with *S*_*T*_[**X**]. Among the rare trajectories corresponding to the phase transition, the least improbable one minimises the action. This optimal path dominates the statistics of the transition in the low-noise limit, effectively governing the most likely mechanism by which the system escapes from metastability. This trajectory (instanton) is known as the MLP for the transition from **X**_1_ to **X**_2_.

Since **X**_1_ and **X**_2_ represent thermodynamic equilibrium states (with zero velocity), they correspond to critical points of the functional ${{\mathcal{H}}}$ in Eq. (8), which are asymptotically stable. These minima identify distinct basins of attraction in trajectory space, separated by a saddle point associated with the critical nucleus configuration. Let us call *T*^⋆^ the time when the path crosses the separatrix. The optimal path consists of two dynamically distinct but continuous segments: a fluctuation-driven segment from **X**_1_ to the saddle point, for −*T *≤ *t *≤ *T*^⋆^, and a (substantially) deterministic relaxation from the saddle to **X**_2_ for *T*^⋆ ^≤ *t *≤ *T*. The former corresponds to the nucleation phase proper, during which thermal noise drives the system across the barrier; the latter describes the spontaneous expansion of the supercritical bubble toward the stable vapour state.

This picture naturally suggests a decomposition of the total action into two distinct contributions.

22 $$\begin{array}{l}{S}_{T}[{{\bf{X}}}]=\frac{1}{2}{\int }_{-T}^{{T}^{\star }}{\left\Vert {\partial }_{t}{{\bf{X}}}+{{\mathcal{T}}}\left[{{\bf{X}}}\right]+{{\mathcal{A}}}\frac{\delta {{\mathcal{H}}}}{\delta {{\bf{X}}}}\right\Vert }_{{{{\mathcal{A}}}}^{+}}^{2}{{\rm{d}}}t\\ +\frac{1}{2}{\int }_{{T}^{\star }}^{T}{\left\Vert {\partial }_{t}{{\bf{X}}}+{{\mathcal{T}}}\left[{{\bf{X}}}\right]+{{\mathcal{A}}}\frac{\delta {{\mathcal{H}}}}{\delta {{\bf{X}}}}\right\Vert }_{{{{\mathcal{A}}}}^{+}}^{2}{{\rm{d}}}t\\ ={S}_{T}^{1}[{{\bf{X}}}]+{S}_{T}^{2}[{{\bf{X}}}].\end{array}$$

As shown in Supplementary Note 8, one can write

23 $${S}_{T}^{1}[{{\bf{X}}}]={\int }_{-T}^{{T}^{\star }}\left(\frac{1}{2}{\left\Vert {\partial }_{t}{{\bf{X}}}+{{\mathcal{T}}}\left[{{\bf{X}}}\right]-{{{\mathcal{A}}}}^{{\dagger} }\frac{\delta {{\mathcal{H}}}}{\delta {{\bf{X}}}}[{{\bf{X}}}]\right\Vert }_{{{{\mathcal{A}}}}^{+}}^{2}+2\frac{{{\rm{d}}}{{\mathcal{H}}}}{{{\rm{d}}}t}\right){{\rm{d}}}t,$$

hence, the action functional takes the form

24 $$\begin{array}{l}{S}_{T}[{{\bf{X}}}]=\frac{1}{2}{\int }_{-T}^{{T}^{\star }}{\left\Vert {\partial }_{t}{{\bf{X}}}+{{\mathcal{T}}}\left[{{\bf{X}}}\right]-{{{\mathcal{A}}}}^{{\dagger} }\frac{\delta {{\mathcal{H}}}}{\delta {{\bf{X}}}}[{{\bf{X}}}]\right\Vert }_{{{{\mathcal{A}}}}^{+}}^{2}\,{{\rm{d}}}t\\ +\frac{1}{2}{\int }_{{T}^{\star }}^{T}{\left\Vert {\partial }_{t}{{\bf{X}}}+{{\mathcal{T}}}\left[{{\bf{X}}}\right]+{{\mathcal{A}}}\frac{\delta {{\mathcal{H}}}}{\delta {{\bf{X}}}}[{{\bf{X}}}]\right\Vert }_{{{{\mathcal{A}}}}^{+}}^{2}{{\rm{d}}}t\\ +2\left({{\mathcal{H}}}[{{\bf{X}}}({{\bf{x}}},{T}^{\star })]-{{\mathcal{H}}}[{{\bf{X}}}({{\bf{x}}},-T)]\right).\end{array}$$

Since, **X**_1_ = (*ρ*_L_, **0**) and **X**_2_ = (*ρ*_V_, **0**) are two (asymptotically) stable minima of ${{\mathcal{H}}}$, the lower bound of *S*_*T*_[**X**] can be found by minimising the functional,

25 $$\begin{array}{l}{S}_{\infty }[{{\bf{X}}}]=\frac{1}{2}{\int }_{-\infty }^{{T}^{\star }}{\left\Vert {\partial }_{t}{{\bf{X}}}+{{\mathcal{T}}}\left[{{\bf{X}}}\right]-{{{\mathcal{A}}}}^{{\dagger} }\frac{\delta {{\mathcal{H}}}}{\delta {{\bf{X}}}}\right\Vert }_{{{{\mathcal{A}}}}^{+}}^{2}\,{{\rm{d}}}t\\ +\frac{1}{2}{\int }_{{T}^{\star }}^{+\infty }{\left\Vert {\partial }_{t}{{\bf{X}}}+{{\mathcal{T}}}\left[{{\bf{X}}}\right]+{{\mathcal{A}}}\frac{\delta {{\mathcal{H}}}}{\delta {{\bf{X}}}}\right\Vert }_{{{{\mathcal{A}}}}^{+}}^{2}{{\rm{d}}}t\\ +2\left({\Omega }^{\star }-{\Omega }_{1}\right),\end{array}$$

with

26 $$\begin{array}{l}{\Omega }^{\star }=\Omega [{\rho }^{\star },\theta ]={\int }_{{{\mathcal{D}}}}{f}_{{{\rm{b}}}}({\rho }^{\star },\theta )+\frac{\lambda }{2}| \nabla {\rho }^{\star }{| }^{2}\,-{\mu }_{{{\rm{eq}}}}{\rho }^{\star }{{\rm{d}}}V,\\ {\Omega }_{1}=\Omega [{\rho }_{{{\rm{L}}}},\theta ]={\int }_{{{\mathcal{D}}}}{f}_{{{\rm{b}}}}({\rho }_{{{\rm{L}}}},T)-{\mu }_{{{\rm{eq}}}}{\rho }_{{{\rm{L}}}}\,{{\rm{d}}}V.\end{array}$$

The MLP is the trajectory connecting the two minima **X**_1_ and **X**_2_ formed by the two branches satisfying

27 $${\partial }_{t}{{\bf{X}}}=-{{\mathcal{T}}}\left[{{\bf{X}}}\right]+{{{\mathcal{A}}}}^{{\dagger} }\frac{\delta {{\mathcal{H}}}}{\delta {{\bf{X}}}}\,\,\,\,\,\,\,\,\,\,\,t\in [-\infty ,{T}^{\star }],$$

and

28 $${\partial }_{t}{{\bf{X}}}=-{{\mathcal{T}}}\left[{{\bf{X}}}\right]-{{\mathcal{A}}}\,\frac{\delta {{\mathcal{H}}}}{\delta {{\bf{X}}}}\,\,\,\,\,\,\,\,\,\,\,t\in [{T}^{\star },+\infty ],$$

respectively. The associated probability of the transition is

29 $${{{\bf{P}}}}_{{{\rm{pc}}}} \sim \exp \left(-\frac{\Delta {\Omega }^{\star }}{{k}_{{{\rm{B}}}}\theta }\right),$$

with Δ*Ω*^⋆^ = *Ω*^⋆^ − *Ω*_1_. The two segments of the transition path — the nucleation phase from **X**_1_ to the saddle point **X**^⋆^ = (*ρ*^⋆^, 0) and the subsequent bubble expansion toward **X**_2_ — share the same hydrodynamic structure, denoted by the contribution ${{\mathcal{T}}}$. This term includes the conservative components of the dynamics, which comprise the conservation laws of mass and momentum, in addition to the effects of viscous dissipation. While these contributions are structurally identical along both segments of the transition path, the sign of the viscous term is effectively reversed: it plays the role of a dissipative sink during the relaxation toward the stable vapour state, whereas it functions as a source of energy during the nucleation process.

This reversal highlights a fundamental aspect of fluctuation-induced phase transitions. Along the nucleation branch, the viscous term in Eq. (27) acts as an effective injector of energy. The physical interpretation is that thermal noise should provide twice the energy physically dissipated by viscosity, resulting in a positive energy contribution of the effective (anti-)viscous term appearing in Eq. (27). In doing so, it counteracts the natural damping of the system, inducing the crossing of the nucleation barrier separating the metastable and stable states. This behaviour characterises a fluctuation-driven, anti-hydrodynamic regime, wherein dissipative processes are effectively reversed. Such dynamics are consistent with the structure predicted by large deviation theory for optimal fluctuation paths^39,46,51^.

Eventually, this analysis leads to the conclusion that deterministic dynamics with a reversed viscous force provide the branch of the MLP connecting the metastable liquid with the critical bubble. In fact, Eq. (27), expressed in the original variables, reads

30 $${\partial }_{t}\rho =-\nabla \cdot \left(\rho {{\bf{v}}}\right),$$

31 $${\partial }_{t}{{\bf{v}}}=-({{\bf{v}}}\cdot \nabla ){{\bf{v}}}-\nabla \mu -\frac{{\eta }_{1}}{\rho }{\nabla }^{2}{{\bf{v}}}-\left(\frac{{\eta }_{2}}{\rho }+\frac{{\eta }_{1}}{3\rho }\right)\nabla \nabla \cdot {{\bf{v}}}.$$

The solution is appropriately obtained after enforcing the following transformations

$$t\to -\tau \,\,\,\,\,\,{{\bf{v}}}\to -{{\bf{w}}}\,\,\,\,\,\,\rho \to \rho$$

resulting in

32 $${\partial }_{\tau }\rho =-\nabla \cdot \left(\rho {{\bf{w}}}\right),$$

33 $${\partial }_{\tau }{{\bf{w}}}=-({{\bf{w}}}\cdot \nabla ){{\bf{w}}}-\nabla \mu +\frac{{\eta }_{1}}{\rho }{\nabla }^{2}{{\bf{w}}}+\left(\frac{{\eta }_{2}}{\rho }+\frac{{\eta }_{1}}{3\rho }\right)\nabla \nabla \cdot {{\bf{w}}}.$$

In this form, it recovers the (deterministic) Capillary Navier-Stokes (CNS) equations, with the reversed evolution running in the opposite direction, starting from the final state **X**^⋆^ (saddle point) to the initial state **X**_1_. Conversely, after reaching the saddle point, the dynamics spontaneously follow the (standard) deterministic hydrodynamic equations.

### MLP simulations

To identify the MLP, we first locate the transition state on the Landau free energy landscape using the Gentlest Ascent Dynamics (GAD) method^52^, which allows us to converge to the saddle point along the unstable manifold. This critical configuration is then embedded into a deterministic solver for the compressible CNS equations, Eqs. (2) and (3)). To trigger the two relaxations (from **X**^⋆^ to **X**_1/2_), we slightly perturb the system along its unstable eigenmode in both directions of the unstable manifold-towards the metastable basin (liquid) and towards the stable one (vapour). Once the relaxation phase is obtained, we recover the nucleation branch by reversing the time variable and the sign of the velocity field. The expansion branch, on the other hand, is obtained by integrating the equations (Eqs. (2) and (3))) forward in time. The nucleation pathway refers to pure water at atmospheric pressure and at a temperature of 302 °C and density *ρ* = 684.2 kg m^−3^. The initial configuration was extracted from the GAD described in the next section. The simulation domain consists of 8000 grid points over a physical length *L*_ref_ = 1000 nm; The time step used in the simulations is Δ*t* = 3.82 × 10^−15^ s. The code integrates the Eqs. (2) and (3)) in spherically symmetry (see ref. ^53^ for details) with boundary conditions enforcing homogeneous Neumann conditions for the density at both *r* = 0 and *r* = *R*, zero velocity at the origin (*v*(0) = 0), and zero derivative of velocity at the outer boundary (∂_*r*_*v*(*R*) = 0). In practice, the choice of boundary conditions at *r* = *R* has a negligible effect due to the large domain size. All thermophysical properties of water, including density, viscosity, and surface tension, were obtained from the IAPWS-95 formulation^20^. Time integration is carried out using a second-order Runge-Kutta explicit scheme^54^, also well-suited for stochastic dynamics.

### Nucleation rate

The nucleation rate is defined as *J* = 1/(*τV*), where *V* is the system volume while *τ* is the mean first passage time, i.e. the characteristic time required for a vapour bubble to nucleate in a metastable liquid. In simple reversible thermodynamic systems-overdamped diffusion on an energy landscape (Brownian motion in a potential)-the mean waiting time *τ* is estimated by classical Kramers theory^55,56^. For the dynamics considered here, we use the Langer-Bouchet-Reygner generalisation of the Eyring-Kramers transition-rate formula^48,57^.

34 $$\tau \sim \frac{2\pi }{\kappa }{\left(\frac{| \det {H}^{\star }| }{\det {H}_{1}}\right)}^{1/2}\exp \left(\frac{\Delta {\Omega }^{\star }}{{k}_{{{\rm{B}}}}\theta }\right),$$

where *κ* is the (unique) positive eigenvalue of the CNS equations linearised around **X**^⋆^, *H*^⋆^ and *H*_1_ are the Hessian of the Hamiltonian, $H={\delta }^{2}{{\mathcal{H}}}/(\delta {{\bf{X}}}\otimes \delta {{\bf{X}}})$, evaluated at **X**^⋆^ and **X**_1_, respectively.

The ratio of the two functional determinants has been evaluated by Langer and Turski^57^

35 $${\left(\frac{\det {H}_{1}}{| \det {H}^{\star }| }\right)}^{1/2}\simeq \frac{2}{3\sqrt{3}}\left(\frac{V}{{\ell }^{3}}\right){\left(\frac{\sigma {\ell }^{2}}{{k}_{{{\rm{B}}}}\theta }\right)}^{3/2}{\left(\frac{2\sigma }{\ell \Delta p}\right)}^{4},$$

with *ℓ* the density correlation length, defined as $\ell =\sqrt{(\lambda {\rho }_{{{\rm{L}}}})/{c}_{\theta }^{2}}$,^58^. The dynamical prefactor *κ* entering the nucleation rate has been evaluated for droplets^57,59^. Here, we derive the corresponding expression for bubbles based on RP dynamics. By linearising the CNS equations around the critical profile, the density perturbation grows along the unstable mode as $\delta \rho ({{\bf{x}}},t)=\rho ({{\bf{x}}},t)-{\rho }^{\star }({{\bf{x}}}) \sim \exp (\kappa t)$. Assuming spherically symmetric motion, *δρ* can be expressed in terms of the interface displacement *ΔR*(*t*), which for small deviations is approximated as *δρ* ~ − ∂*ρ*^⋆^(*r*)/∂*r* *ΔR*(*t*), with *r* the radial coordinate. The escape rate from the saddle point is therefore estimated as from the unstable mode $\Delta R(t) \sim \exp (\kappa t)$. To estimate *κ*, we focus on the overdamped limit and show that the proposed procedure provides an excellent approximation to the numerical eigenvalue obtained both from the CNS dynamics and from the full RP equation, see Supplementary Note 7. We pursue this route also to obtain a compact, readily usable nucleation-rate expression for applications. For an incompressible liquid of density *ρ*_L_ and dynamic viscosity *η*_1_, assuming isothermal conditions with bubble interior pressure *p*_b_ ≈ *p*_V_ and *Δp* = *p*_V_ − *p*_L_ > 0 constant, the overdamped RP equation reads

36 $$\frac{4{\eta }_{1}}{{\rho }_{{{\rm{L}}}}R}\,\frac{{{\rm{d}}}R}{{{\rm{d}}}t}+\frac{2\sigma }{{\rho }_{{{\rm{L}}}}R}-\frac{\Delta p}{{\rho }_{{{\rm{L}}}}}=0,$$

the critical radius is *R*^⋆^ = 2*σ*/*Δp*. Let *R*(*t*) = *R*^⋆^ + *ΔR*(*t*), equation linearised version of Eq. (36) reads

37 $$\frac{{{\rm{d}}}\Delta R}{{{\rm{d}}}t}-\frac{\Delta p}{4{\eta }_{1}}\,\Delta R=0.$$

Therefore, the dynamical prefactor in the overdamped limit is

38 $$\kappa =\frac{\Delta p}{4\,{\eta }_{1}}\,.$$

Since *J* = 1/(*τV*), by using Eqs. (35) and(38)) we get the expression reported in Eq. (1).

### GAD simulations

We seek the saddle point of the free energy functional *Ω*[*ρ*(***x***), *θ*], without focusing on the specific reaction pathway. A standard gradient descent (Allen Cahn Dynamics),

39 $${\partial }_{t}\rho =-\frac{\delta \Omega }{\delta \rho }$$

cannot reach the saddle point, as it is unstable. Instead, we adopt the GAD^52^, specifically its simplified version^60^, which identifies the saddle point through a coupled dynamics:

40 $${\partial }_{t}\rho =-\frac{\delta \Omega }{\delta \rho }+2\frac{\left(\frac{\delta \Omega }{\delta \rho },u\right)}{\left(u,u\right)}\,u,$$

41 $${\partial }_{t}u=-\frac{{\delta }^{2}\Omega }{\delta {\rho }^{2}}u+\left(u,\frac{{\delta }^{2}\Omega }{\delta {\rho }^{2}}\right)\,u.$$

The field *u*(**x**, *t*) evolves according to a Rayleigh quotient gradient flow^61^. At steady state, *u* becomes the eigenfunction associated with the minimum and only negative eigenvalue of the Hessian (${\delta }^{2}\Omega /\delta {\rho }^{2}={c}_{\theta }^{2}/\rho -\lambda {\nabla }^{2}$), which identifies the unstable direction of the system. The equation for *ρ* at steady state therefore converges to the saddle point *ρ*^⋆^(**x**). The GAD simulations are performed using the same discretisation as the dynamic simulations.

### MEP simulations

The MEP is defined as a continuous sequence of fields *ρ*(*s*, **x**) satisfying

42 $${\left(\frac{\delta \Omega }{\delta \rho }\right)}^{\perp }\left[\rho (s,{{\bf{x}}})\right]=0,$$

where ⊥ indicates the projection of the functional derivative orthogonal to the path direction:

43 $${\left(\frac{\delta \Omega }{\delta \rho }\right)}^{\perp }=\frac{\delta \Omega }{\delta \rho }-\frac{1}{\left(\frac{\partial \rho }{\partial s},\frac{\partial \rho }{\partial s}\right)}\frac{\partial \rho }{\partial s}\left(\frac{\delta \Omega }{\delta \rho },\frac{\partial \rho }{\partial s}\right).$$

Following the String Method^62^, the path is discretised into a finite number of fields *ρ*^*s*^(**x**), referred to as images, which form the so-called string. Starting from an initial guess-typically a linear interpolation between the metastable liquid and the vapour-the string evolves in a pseudo-time $\widetilde{t}$ using a steepest descent update:

44 $${\partial }_{\tilde{t}}{\rho }^{s}={\mu }_{{{\rm{eq}}}}-\mu ({\rho }^{s})+\lambda {\nabla }^{2}{\rho }^{s}.$$

A staggered spatial discretisation is adopted, with spherical symmetry. The equation is integrated in time via a forward Euler scheme. After each time step, the images are redistributed to maintain uniform arclength along the string^62^, see also refs. ^63,64^ for application to other DI (Ginzburg-Landau) functionals,

45 $$\delta s=\left|\left|{\rho }^{s+1}({{\bf{x}}})-{\rho }^{s}({{\bf{x}}})\right|\right|=\,{{\rm{constant}}}\,\,.$$

This evolution and redistribution loop is iterated until convergence, i.e., when the energy profile *Ω*(*s*) becomes stationary. The string starts at *s* = 0 with the metastable homogeneous liquid, and ends at *s* = 1 with the vapour phase at the same chemical potential. To enhance convergence, the initial configuration includes a large, overcritical bubble-roughly 75% of the domain radius-ensuring faster formation of a vapour-like region. Intermediate images are initially linearly interpolated.

Upon convergence, the resulting MEP reveals the nucleation mechanism. In all simulations, the path is resolved using 400 images, with refinement of the early stage of the process conducted using Allen-Cahn dynamics relaxation. The spatial discretisation space Δ = 0.125 nm on a domain of length *L* = 1000 nm. The pseudo-time step was selected as $\Delta \widetilde{t}=0.001$.

### CLLNS simulations

We model spontaneous vapour nucleation in a metastable liquid using the fluctuating hydrodynamics (FH) framework extended to multiphase systems^16^. This approach builds on Landau and Lifshitz’s theory, incorporating thermal noise in the stress tensor and energy flux-derived from a fluctuation-dissipation theorem-to ensure sampling of the Einstein-Boltzmann distribution^18,24,65–70^.

We employ a coarse-grained, spherically symmetric version of the model, obtained by averaging the full 3D equations over radial shells. The resulting system depends only on the radial distance from the cluster centre^16^. The stochastic evolution equations are reported below for clarity:

46 $$\begin{array}{l}{\partial }_{t}\rho =-\frac{1}{{r}^{2}}{\partial }_{r}({r}^{2}\rho {v}_{r}),\\ \rho\, \partial_t {v}_{r}=-\rho {\partial }_{r}\left(\frac{{v}_{r}^{2}}{2}+\frac{\delta \Omega }{\delta \rho }\right)+\frac{1}{{r}^{2}}{\partial }_{r}({r}^{2}{\Sigma }_{rr}^{v})-\frac{2}{r}{\Sigma }_{\varphi \varphi }^{v}\\ +\frac{1}{{r}^{2}}{\partial }_{r}\left(\beta r\,\xi (r,t)\right)+\frac{\beta }{{r}^{2}}\xi (r,t),\end{array}$$

with

47 $${\mathop{\sum }\limits_{rr}^{v}}=\frac{4}{3}{\eta }_{1}\left(\frac{\partial {v}_{r}}{\partial r}-\frac{{v}_{r}}{r}\right)\,\,{{\mathop{\sum }\limits_{\varphi \varphi }^{v}}}=\frac{2}{3}{\eta }_{1}\left(\frac{{v}_{r}}{r}-\frac{\partial {v}_{r}}{\partial r}\right)\,,$$

$\beta =\sqrt{(2{k}_{{{\rm{B}}}}T)/(3\pi {\eta }_{1})}$ and $\langle \xi (r,t)\xi (r^{\prime} ,t^{\prime} )\rangle =\delta (r-r{\prime} )\delta (t-t{\prime} )$. The simulations were performed using the same parameters as the deterministic ones, but with an initial condition consisting of a uniform metastable state with zero velocity, i.e. *θ* = 302 °C and density *ρ* = 684.2 kg m^−3^, *v*_*r*_ = 0 m s^−1^.

## Supplementary information

Transparent Peer Review file

Supplementary Information

## Data Availability

The final data supporting this research are available in the open Zenodo repository of the E-NUCL project: https://zenodo.org/communities/e-nucl10.5281/zenodo.19187426.

## Code availability

Computer codes used for the calculations and the generation of figures are available in the open Zenodo repository of the E-NUCL project: https://zenodo.org/communities/e-nucl10.5281/zenodo.19187426.
