# Supplementary material for: Hydrodynamical pathways in the phase change of real fluids
Source: Commun Phys. 2026 Apr 27;9(1):220. doi: 10.1038/s42005-026-02639-y (PMC13323055; doi:10.1038/s42005-026-02639-y)
Supplement: Supplementary file 2 — Supplementary Information [file 42005_2026_2639_MOESM2_ESM.pdf]

Supplementary Information on "Hydrodynamical pathways in the phase change of real fluids".

Mirko Gallo, Filippo Occhioni, Riccardo Daniele, Carlo Massimo Casciola

Supplementary Note 1: Equations of State

Multiparametric equations of state are functions that describe the thermodynamic properties of a real fluid, either as a single-component or multicomponent substance. Once a specific functional form is chosen, a large set of experimental data is fitted by adjusting appropriate free parameters. This process yields an expression in the form of a thermodynamic potential, such as the Helmholtz free energy, as a function of reduced state variables, namely the reduced density $\delta = \rho/\rho_c$ and the inverse reduced temperature $\tau = T_c/T$. This expression provides a complete description of the fluid's thermodynamic behaviour, from which all relevant properties can be derived via appropriate thermodynamic differentiation. For a single-component fluid, the Helmholtz free energy per unit mass is typically expressed as the sum of an ideal part ϕ_0 and a residual part ϕ_r, which accounts for deviations from ideal behaviour:

$$\frac{\hat{f}(\rho, T)}{RT} = \phi(\delta, \tau) = \phi^0(\delta, \tau) + \phi^r(\delta, \tau) \tag{S1}$$

where R is the specific gas constant. The behaviour of water is modelled using the IAPWS-95 formulation [1], while the thermodynamic properties of nitrogen and helium IV are taken from the GERG-2008 [2] equation of state. The specific functional forms used, along with the fitted parameter values, are presented comprehensively in the following subsections. For further details, the reader is referred to the original works.

Nitrogen and Helium IV - GERG2008

The GERG-2008 [2] equation of state was developed to accurately represent the thermodynamic properties of natural gases and a wide variety of other gas mixtures. The functional forms for the ideal and residual contributions are shown in Equations (S2), (S3). The respective coefficients for the two fluids are reported in Supplementary Tables 3, 4. The symbol R^* denotes a reference ideal gas constant whose value is $R^* = 8.314510$ J mol^{-1} K^{-1}.

$$\phi^{\mathrm{o}}(\delta, \tau) = \ln(\delta) + \frac{R^*}{R}\left[n_1^{\mathrm{o}} + n_2^{\mathrm{o}}\tau + n_3^{\mathrm{o}}\ln(\tau) + \sum_{k=4,6} n_k^{\mathrm{o}} \ln\left(\left|\sinh\left(\vartheta_k^{\mathrm{o}}\tau\right)\right|\right) + \sum_{k=5,7} n_k^{\mathrm{o}} \ln\left(\cosh\left(\vartheta_k^{\mathrm{o}}\tau\right)\right)\right] \tag{S2}$$

$$\phi^{\mathrm{r}}(\delta, \tau) = \sum_{k=1}^{K_{\mathrm{Pol}}} n_k \delta^{d_k} \tau^{t_k} + \sum_{k=K_{\mathrm{Pol}}+1}^{K_{\mathrm{Pol}}+K_{\mathrm{Exp}}} n_k \delta^{d_k} \tau^{t_k} \exp(-\delta^{c_k}) \tag{S3}$$

Fluid	T_c (K)	P_c (MPa)	ρ_c (kg/m³)
Nitrogen	126.192	3.396	313.3
Helium	5.195	0.227	69.6
Water	647.096	22.064	322.0

Supplementary Table 1. Critical temperature, pressure and density of Nitrogen, Helium and Water

Water - IAPWS95

The IAPWS-95 [1] is the most accurate equation of state for water, capable of reproducing its thermodynamic properties over a wide range of temperatures and densities. The following expressions give the ideal and residual parts of the Helmholtz free energy, while the corresponding coefficients are listed in Supplementary Tables 5, 6.

$$\phi^{\mathrm{o}} = \ln(\delta) + n_1^{\mathrm{o}} + n_2^{\mathrm{o}}\tau + n_3^{\mathrm{o}}\ \ln(\tau) + \sum_{i=4}^{8} n_i^{\mathrm{o}}\ \ln\left[1 - \exp(-\gamma_i^{\mathrm{o}}\tau)\right] \tag{S4}$$

$$\phi^{\mathrm{r}} = \sum_{i=1}^{7} n_i\delta^{d_i}\tau^{t_i} + \sum_{i=8}^{51} n_i\delta^{d_i}\tau^{t_i}\exp\left(-\delta^{c_i}\right) + \sum_{i=52}^{54} n_i\delta^{d_i}\tau^{t_i}\exp\left[-\alpha_i\left(\delta-\varepsilon_i\right)^2 - \beta_i\left(\tau-\gamma_i\right)^2\right] + \sum_{i=55}^{56} n_i\Delta^{a_i}\delta\,\psi \tag{S5}$$

with

$$\begin{aligned} \Delta &= \theta^2 + B_i\left[(\delta-1)^2\right]^{a_i} \\ \theta &= (1-\tau) + A_i\left[(\delta-1)^2\right]^{\left(\frac{1}{2\beta_i}\right)} \\ \psi &= \exp\left[-C_i(\delta-1)^2 - D_i(\tau-1)^2\right] \end{aligned} \tag{S6}$$

Supplementary Note 2: EoS Correction

The van der Waals square-gradient approximation provides a method to represent the coexistence of multiple phases in a fluid. To apply this approach, it is necessary to have an equation of state capable of describing not only the fluid under stable conditions but also under metastable and unstable states. The advantage of multiparametric equations of state is that they are derived by fitting experimental data that also cover metastable fluid states, enabling an accurate and quantitative description of the metastable regime. In the instability region, however, experimental measurements are not feasible, as the fluid undergoes spinodal decomposition. As a result, the predictions of the multiparametric equations of state in this region are physically unreliable. Specifically, quantities such as energy, pressure, and chemical potential exhibit strong oscillations, commonly referred to as Maxwell loops [3], which lead to the appearance of non-physical stationary states and can compromise the stability and convergence of numerical methods. To address this issue, the correction method proposed in [4] has been applied. The Helmholtz free energy per unit mass is expressed as a piecewise-defined function:

$$\hat{f}^{corr}(\rho,T) = \begin{cases} \hat{f}^{eos}(\rho,T), & \rho \le \rho_V^{spin}(T) \cup \rho \ge \rho_L^{spin}(T) \\ \hat{f}^{mod}(\rho,T), & \rho_V^{spin}(T) < \rho < \rho_L^{spin}(T) \end{cases} \tag{S7}$$

where ρ_v^{spin} and ρ_l^{spin} denote the densities along the spinodal curve, the line that separates the metastable region from the unstable one. By enforcing continuity and differentiability of the energy across the two branches of the function, and recalling that the spinodal curve satisfies the condition $\partial p/\partial\rho = 0$, any modified equation of state $\hat{f}_{mod}$ is required to meet the following conditions:

$$
\begin{cases}
p^{mod}\left(\rho_V^{spin}\right) = p\left(\rho_V^{spin}\right) \\
p^{mod}\left(\rho_L^{spin}\right) = p\left(\rho_L^{spin}\right) \\
\dfrac{\partial p^{mod}}{\partial \rho}\left(\rho_V^{spin}\right) = 0 \\
\dfrac{\partial p^{mod}}{\partial \rho}\left(\rho_L^{spin}\right) = 0 \\
\hat{f}^{mod}\left(\rho_V^{spin}\right) = \hat{f}\left(\rho_V^{spin}\right) \\
\hat{f}^{mod}\left(\rho_L^{spin}\right) = \hat{f}\left(\rho_L^{spin}\right)
\end{cases}
\tag{S8}
$$

The functional forms used for the free energy and for the pressure are the following

$$
\begin{aligned}
\hat{f}^{\mathrm{mod}} &= \int \frac{p^{\mathrm{mod}}}{\rho^2}\, d\rho + F \\
p^{\mathrm{mod}} &= A\rho^3 + B\rho^2 + C\rho + D + E\exp(\rho)\rho^2
\end{aligned}
\tag{S9}
$$

where A, B, C, D, E and F are constants chosen to satisfy the constraints described above. The specific functional form adopted does not appear to significantly affect macroscopic observables such as the nucleation rates [4]. Supplementary Figure 1 shows the GERG-2008 pressure correction for Nitrogen at $-160°C$.

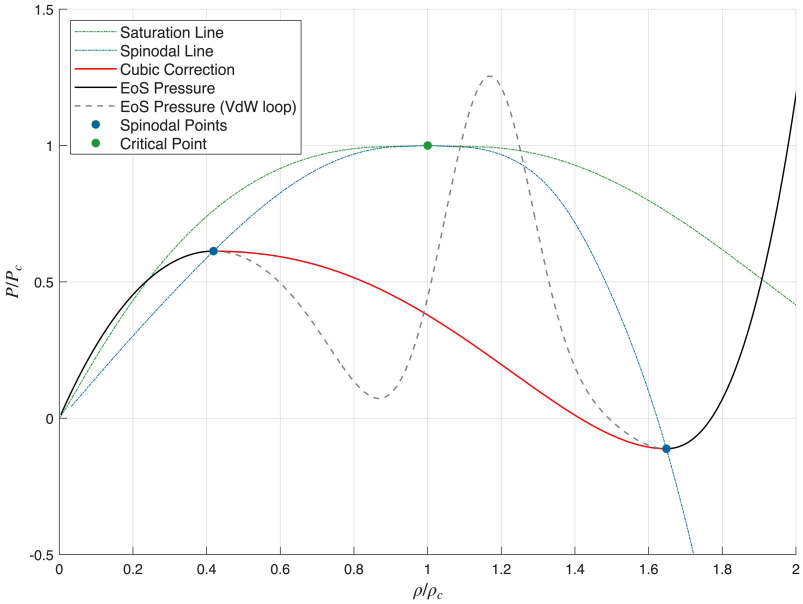

Supplementary Figure 1. Schematic of the Maxwell loop correction for the multiparametric Equation of State. In solid black, the EoS isotherm curve is kept unchanged; in dashed black, the Van der Waals loop of the EoS isotherm; in red the corrected version. Additionally, the spinodal and binodal lines are shown in blue and green, respectively.

Supplementary Note 3: Surface Tension

In order to properly calibrate the diffuse interface model, the surface tension of the planar interface needs to be known. The surface tension correlations used in this work [5] are based on a version of the NIST REFPROP model [6]. The general expression is reported below, while the parameters used are listed in Supplementary Table 2.

$$\sigma(T) = \sum_{i=0}^{k-1} \sigma_i \left(1 - \frac{T}{T_c}\right)^{n_i} \tag{S10}$$

Fluid	σ_0	n_0	σ_1	n_1	σ_2	n_2
Nitrogen	$0.028\,98$	1.246				
Helium	$0.000\,465\,6$	1.040	$0.001\,889$	2.468	$-0.002\,006$	2.661
Water	-0.1306	2.471				

Supplementary Table 2. Surface Tension parameters of Nitrogen, Helium and Water taken from Ref. [5]

Supplementary Note 4: Capillary coefficient

Once an equation of state and the surface tension of the planar interface are known, the capillary coefficient can be estimated using the following relation [4]:

$$\lambda(T) = \frac{\sigma^2(T)}{\left(\int_{\rho_V^{\mathrm{sat}}}^{\rho_L^{\mathrm{sat}}} \sqrt{2\,\Delta\omega}\, d\rho\right)^2} \tag{S11}$$

where $\Delta\omega = \rho f - \mu^{sat}\rho + p^{sat}$ denotes the difference in grand potential density. The capillary coefficient is treated as a fitting parameter to reproduce the surface tension at a fixed temperature and has no explicit connection to the fluid's direct correlation function. The values of the dimensionless capillary coefficient ($\lambda_{ref} = P_c L_{ref}^2/\rho_c$ with $L_{ref} = 1\,nm$ and P_c , ρ_c as reported in Table 1) as a function of temperature for the fluids under study are shown in Figure 2. Since the equations of state are mean-field, they do not accurately reproduce the scaling behaviour near the critical point. Similarly, the expression for the surface tension is derived from fitting experimental data and does not account for the proper critical exponents. Therefore, simulations conducted too close to the critical point may yield inaccurate results and have been avoided.

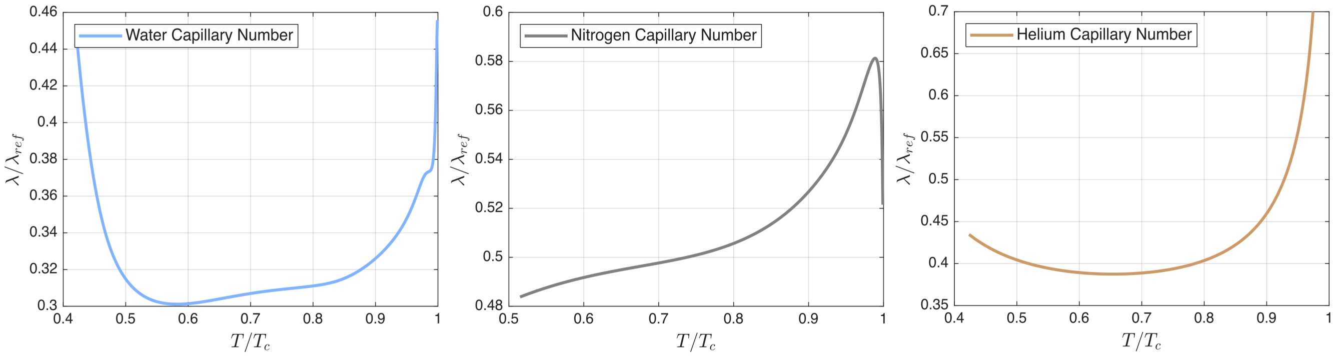

Supplementary Figure 2. Capillary coefficient profiles. The capillary coefficient λ as a function of temperature is reported for the three fluids analyzed in the study: water in light blue, nitrogen in gray, helium in brown.

Supplementary Note 5: Density Profile MEP

This section presents the Minimum Energy Path for the nucleation of a vapour bubble in superheated water. The sequence of density profiles constituting the MEP has the saddle-point configuration highlighted in black. Supercritical (shades of red) and precritical (shades of blue) profiles are also shown. The arrows indicate the direction of progression along the reaction pathway. The thermodynamic conditions are the same as those discussed in the Results section of the main text ($\rho = 684.2 \mathrm{kg/m^3}$ and $\theta = 302°\mathrm{C}$). In all simulations, the path is resolved using 400 images, with refinement of the early stage of the process conducted using Allen-Cahn dynamics relaxation. The spatial discretisation space $\Delta = 0.125\mathrm{nm}$ on a domain of length $L = 100\mathrm{nm}$. The pseudo-time step was selected as $\Delta\tilde{t} = 0.001$.

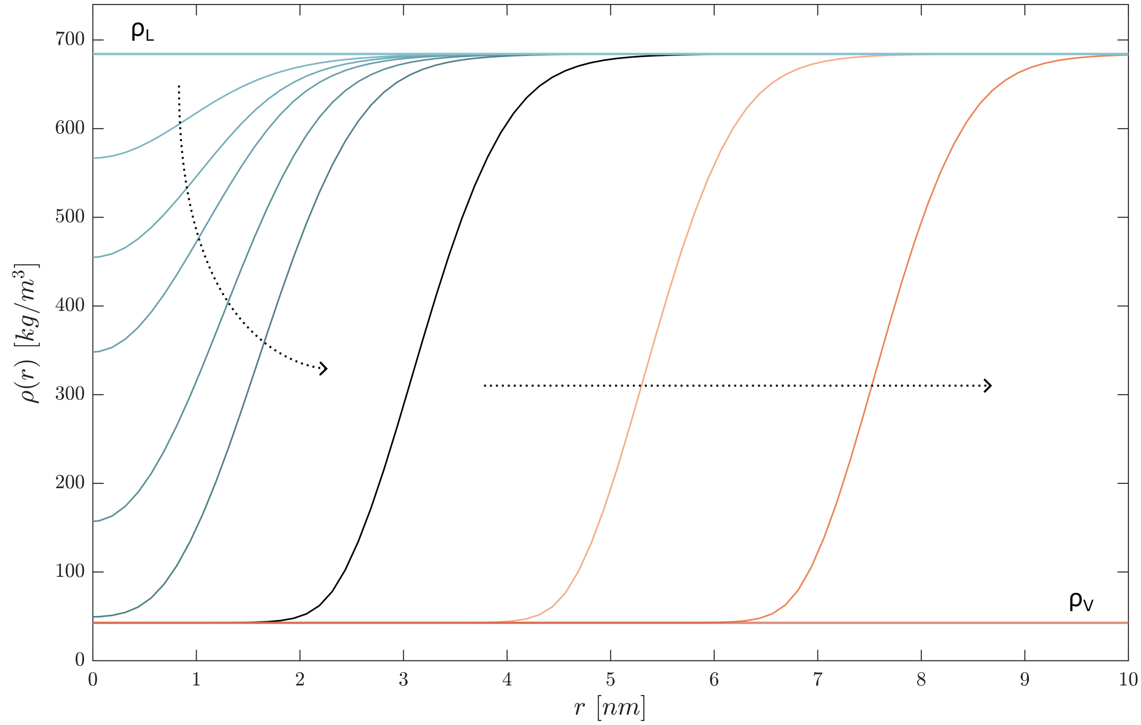

Supplementary Figure 3. Radial density profiles of the bubble nucleation along the Minimum Energy Path. Sequence of radial density profiles for vapour bubble nucleation in superheated water at $\rho = 684.2\,\mathrm{kg/m^3}$ and $\theta = 302°\mathrm{C}$. The critical nucleus (saddle-point configuration) is highlighted in black. Precritical profiles are shown in shades of blue, supercritical profiles in shades of red. Arrows indicate the direction of progression along the reaction pathway.

Supplementary Note 6: EoS Coefficients

k	n_k°	ϑ_k°	k	n_k°	ϑ_k°
		Nitrogen			
1	$11.083\,407\,489$		5	$-0.146\,60$	$-5.393\,067\,706$
2	$-22.202\,102\,428$		6	$0.900\,66$	$13.788\,988\,208$
3	$2.500\,31$		7		
4	$0.137\,32$	$5.251\,822\,620$			
		Helium			
1	$13.628\,409\,737$		5		
2	$-143.470\,759\,602$		6		
3	$1.500\,00$		7		
4					

Supplementary Table 3. Nitrogen and Helium ideal EoS coefficients for Equation (S2) taken from Ref. [2]

k	n_k	c_k	d_k	t_k	k	n_k	c_k	d_k	t_k
				Nitrogen					
1	$0.598\,897\,118\,012\,01$		1	0.125	13	$-0.169\,366\,415\,549\,83$	2	2	4.500
2	$-0.169\,415\,574\,807\,31 \cdot 10^{1}$		1	1.125	14	$0.135\,468\,460\,417\,01$	2	3	4.750
3	$0.245\,797\,361\,917\,18$		2	0.375	15	$-0.330\,667\,120\,953\,07 \cdot 10^{-1}$	2	3	5.000
4	$-0.237\,224\,567\,551\,75$		2	1.125	16	$-0.606\,908\,170\,185\,57 \cdot 10^{-1}$	2	4	4.000
5	$0.179\,549\,187\,151\,41 \cdot 10^{-1}$		4	0.625	17	$0.127\,975\,482\,928\,71 \cdot 10^{-1}$	2	4	4.500
6	$0.145\,928\,757\,202\,15 \cdot 10^{-1}$		4	1.500	18	$0.587\,436\,641\,072\,99 \cdot 10^{-2}$	3	2	7.500
7	$0.100\,080\,659\,362\,06$	1	1	0.625	19	$-0.184\,519\,519\,719\,69 \cdot 10^{-1}$	3	3	14.000
8	$0.731\,571\,153\,855\,32$	1	1	2.625	20	$0.472\,266\,220\,424\,72 \cdot 10^{-2}$	3	4	11.500
9	$-0.883\,722\,723\,363\,66$	1	1	2.750	21	$-0.520\,240\,796\,805\,99 \cdot 10^{-2}$	6	5	26.000
10	$0.318\,876\,602\,467\,08$	1	2	2.125	22	$0.435\,635\,059\,566\,35 \cdot 10^{-1}$	6	6	28.000
11	$0.207\,664\,917\,287\,99$	1	3	2.000	23	$-0.362\,516\,907\,509\,39 \cdot 10^{-1}$	6	6	30.000
12	$-0.193\,793\,154\,541\,58 \cdot 10^{-1}$	1	6	1.750	24	$-0.289\,740\,268\,665\,43 \cdot 10^{-2}$	6	7	16.000
	$(K_{Pol,i} = 6, K_{Exp,i} = 18)$								
				Helium					
1	$-0.455\,790\,240\,067\,37$		1	0.000	7	$0.162\,274\,147\,117\,78 \cdot 10^{-1}$	1	5	0.125
2	$0.125\,163\,907\,549\,25 \cdot 10^{1}$		1	0.125	8	$-0.574\,718\,182\,008\,92 \cdot 10^{-1}$	1	5	1.250
3	$-0.154\,382\,316\,506\,21 \cdot 10^{1}$		1	0.750	9	$0.194\,624\,164\,307\,15 \cdot 10^{-1}$	1	5	2.000
4	$0.204\,674\,897\,072\,21 \cdot 10^{-1}$		4	1.000	10	$-0.332\,956\,801\,230\,20 \cdot 10^{-1}$	2	2	1.000
5	$-0.344\,762\,123\,807\,81$	1	1	0.750	11	$-0.108\,635\,773\,723\,67 \cdot 10^{-1}$	3	1	4.500
6	$-0.208\,584\,595\,127\,87 \cdot 10^{-1}$	1	3	2.625	12	$-0.221\,733\,652\,459\,54 \cdot 10^{-1}$	3	2	5.000
	$(K_{Pol,i} = 4, K_{Exp,i} = 8)$								

Supplementary Table 4. Nitrogen and Helium residual EoS coefficients for Equation (S3) taken from Ref. [2]

i	n_i^o	γ_i^o	i	n_i^o	γ_i^o
		Water			
1	$-8.320\,446\,482\,01$		5	$0.973\,15$	$3.537\,342\,22$
2	$6.683\,210\,526\,8$		6	$1.279\,50$	$7.740\,737\,08$
3	$3.006\,32$		7	$0.969\,56$	$9.244\,377\,96$
4	$0.012\,436$	$1.287\,289\,67$	8	$0.248\,73$	$27.507\,510\,5$

Supplementary Table 5. Water ideal EoS coefficients for Equation (S4) taken from Ref. [1]

i	c_i	d_i	t_i	n_i	i	c_i	d_i	t_i	n_i
				Water					
1		1	-0.5	$0.125\,335\,479\,355\,23\cdot10^{-1}$	27	2	3	10	$0.580\,833\,999\,857\,59$
2		1	0.875	$0.789\,576\,347\,228\,28\cdot10^{1}$	28	2	4	3	$0.499\,691\,469\,908\,06\cdot10^{-2}$
3		1	1	$-0.878\,032\,033\,035\,61\cdot10^{1}$	29	2	4	7	$-0.313\,587\,007\,125\,49\cdot10^{-1}$
4		2	0.5	$0.318\,025\,093\,454\,18$	30	2	4	10	$-0.743\,159\,297\,103\,41$
5		2	0.75	$-0.261\,455\,338\,593\,58$	31	2	5	10	$0.478\,073\,299\,154\,80$
6		3	0.375	$-0.781\,997\,516\,879\,81\cdot10^{-2}$	32	2	6	6	$0.205\,279\,408\,959\,48\cdot10^{-1}$
7		4	1	$0.880\,894\,931\,021\,34\cdot10^{-2}$	33	2	6	10	$-0.136\,364\,351\,103\,43$
8	1	1	4	$-0.668\,565\,723\,079\,65$	34	2	7	10	$0.141\,806\,344\,006\,17\cdot10^{-1}$
9	1	1	6	$0.204\,338\,109\,509\,65$	35	2	9	1	$0.833\,265\,048\,807\,13\cdot10^{-2}$
10	1	1	12	$-0.662\,126\,050\,396\,87\cdot10^{-4}$	36	2	9	2	$-0.290\,523\,360\,095\,85\cdot10^{-1}$
11	1	2	1	$-0.192\,327\,211\,560\,02$	37	2	9	3	$0.386\,150\,855\,742\,06\cdot10^{-1}$
12	1	2	5	$-0.257\,090\,430\,034\,38$	38	2	9	4	$-0.203\,934\,865\,137\,04\cdot10^{-1}$
13	1	3	4	$0.160\,748\,684\,862\,51$	39	2	9	8	$-0.165\,540\,500\,637\,34\cdot10^{-2}$
14	1	4	2	$-0.400\,928\,289\,258\,07\cdot10^{-1}$	40	2	10	6	$0.199\,555\,719\,795\,41\cdot10^{-2}$
15	1	4	13	$0.393\,434\,226\,032\,54\cdot10^{-6}$	41	2	10	9	$0.158\,703\,083\,241\,57\cdot10^{-3}$
16	1	5	9	$-0.759\,413\,770\,881\,44\cdot10^{-5}$	42	2	12	8	$-0.163\,885\,683\,425\,30\cdot10^{-4}$
17	1	7	3	$0.562\,509\,793\,518\,88\cdot10^{-3}$	43	3	3	16	$0.436\,136\,157\,238\,11\cdot10^{-1}$
18	1	9	4	$-0.156\,086\,522\,571\,35\cdot10^{-5}$	44	3	4	22	$0.349\,940\,054\,637\,65\cdot10^{-1}$
19	1	10	11	$0.115\,379\,964\,229\,51\cdot10^{-8}$	45	3	4	23	$-0.767\,881\,978\,446\,21\cdot10^{-1}$
20	1	11	4	$0.365\,821\,651\,442\,041\cdot10^{-6}$	46	3	5	23	$0.224\,462\,773\,320\,06\cdot10^{-1}$
21	1	13	13	$-0.132\,511\,800\,746\,68\cdot10^{-11}$	47	4	14	10	$-0.626\,897\,104\,146\,85\cdot10^{-3}$
22	1	15	1	$-0.626\,395\,869\,124\,54\cdot10^{-9}$	48	6	3	50	$-0.557\,111\,185\,656\,45\cdot10^{-9}$
23	2	1	7	$-0.107\,936\,009\,089\,32$	49	6	6	44	$-0.199\,057\,183\,544\,08$
24	2	2	1	$0.176\,114\,910\,087\,52\cdot10^{-1}$	50	6	6	46	$0.317\,774\,973\,307\,38$
25	2	2	9	$0.221\,322\,951\,675\,46$	51	6	6	50	$-0.118\,411\,824\,259\,81$
26	2	2	10	$-0.402\,476\,697\,635\,28$					

i	c_i	d_i	t_i	n_i	α_i	β_i	γ_i	ε_i
52		3	0	$-0.313\,062\,603\,234\,35\times10^{2}$	20	150	1.21	1
53		3	1	$0.315\,461\,402\,377\,81\times10^{2}$	20	150	1.21	1
54		3	4	$-0.252\,131\,543\,416\,95\times10^{4}$	20	150	1.25	1

i	a_i	b_i	B_i	n_i	C_i	D_i	A_i	β_i
55	3.5	0.85	0.2	$-0.148\,746\,408\,567\,124$	28	700	0.32	0.3
56	3.5	0.95	0.2	$0.318\,061\,108\,784\,144$	32	800	0.32	0.3

Supplementary Table 6. Water residual EoS coefficients for Equation (S5) taken from Ref. [1]

Supplementary Note 7: Dynamical prefactor from the linearized Rayleigh–Plesset dynamics

We aim to compute the dynamical prefactor κ entering Langer's nucleation rate for a vapour bubble. Rather than extracting the unstable eigenvalue of the full capillary Navier–Stokes system, we follow pioneering droplet analyses [7] and adopt a single collective–coordinate description in terms of the bubble radius $R(t)$. In this reduced model, κ is the unstable eigenvalue of the Rayleigh–Plesset dynamics linearised about the critical radius. We pursue this route also to obtain a compact, readily usable nucleation–rate expression for applications. The estimated dynamical prefactor κ and its comparison with the numerical value obtained by linearising the compressible Navier–Stokes (CNS) equations are shown in Supplementary Figure 4.
For an incompressible liquid of density ρ_L and dynamic viscosity η_1, assuming isothermal conditions with bubble interior pressure $p_b \approx p_V$ and $\Delta p = p_V - p_L > 0$ constant, the Rayleigh–Plesset equation reads

$$\rho_L\left(R\ddot{R} + \tfrac{3}{2}\dot{R}^2\right) \;=\; \Delta p \;-\; \frac{2\sigma}{R} \;-\; \frac{4\eta_1}{R}\,\dot{R}, \tag{S12}$$

where σ is the surface tension. The critical radius R_c satisfies

$$R_c = \frac{2\sigma}{\Delta p}. \tag{S13}$$

Let $R(t) = R_c + \Delta R(t)$, equation linearised version of Equation (S12) reads

$$\Delta\ddot{R} \;+\; \frac{4\eta_1}{\rho_L R_c^2}\,\Delta\dot{R} \;-\; \frac{2\sigma}{\rho_L R_c^3}\,\Delta R = 0. \tag{S14}$$

The unstable mode $\Delta R(t) \propto e^{\kappa_+ t}$, with $\kappa_+ > 0$, is

$$\kappa_+ = \frac{1}{2}\left[-\frac{\eta_1\,\Delta p^2}{\rho_L\,\sigma^2} + \sqrt{\frac{\eta_1^2 \Delta p^4}{\rho_L^2 \sigma^4} + \frac{\Delta p^3}{\rho_L\,\sigma^2}}\,\right]. \tag{S15}$$

Within the one-coordinate RP description, the dynamical prefactor entering Langer's rate is identified with this unstable growth rate,

$$\kappa \equiv \kappa_+. \tag{S16}$$

Overdamped limit. Neglecting inertia in the linearised RP, the equation for $\Delta R(t)$ is

$$\frac{4\eta_1}{\rho_L R_c^2}\,\Delta\dot{R} \;-\; \frac{2\sigma}{\rho_L R_c^3}\,\Delta R = 0\,, \tag{S17}$$

and the dynamical prefactor is

$$\kappa_{OD} = \frac{\Delta p}{4\,\eta_1}\,. \tag{S18}$$

Asymptotic solution of the Rayleigh–Plesset dynamics in the large-radius limit.

Starting from the Rayleigh–Plesset equation

$$\rho_L\left(R\ddot{R} + \tfrac{3}{2}\dot{R}^2\right) = \Delta p - \frac{2\sigma}{R} - \frac{4\eta_1\dot{R}}{R}, \tag{S19}$$

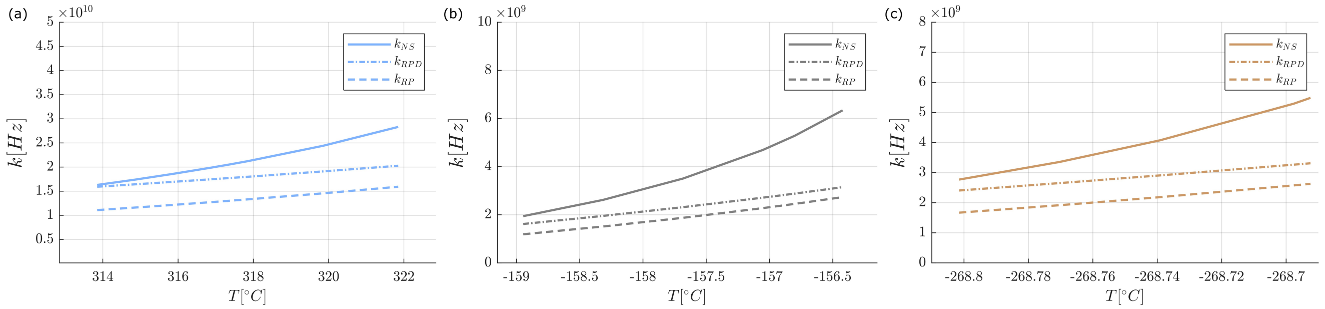

Supplementary Figure 4. Dynamical prefactor profiles as a function of temperature. The solid lines represent the numerical values obtained from the linearised CNS equations, the dash–dotted line represents the theoretical estimate from the overdamped RP model, and the dashed line represents the estimate from the full RP dynamics.

and neglecting the $1/R$ contributions in the large–radius limit, with $\ddot{R} \to 0$ one obtains

$$\tfrac{3}{2}\rho\dot{R}^2 \simeq \Delta p \;\;\Rightarrow\;\; \dot{R} \;\approx\; \sqrt{\tfrac{2}{3}\tfrac{\Delta p}{\rho_L}}\,. \tag{S20}$$

Supplementary Note 8: Derivation of the Eq. 23 of the main text

In this section, we report the procedure adopted to find the instanton minimising the action in Eq. 23. By definition

$$S^1_T[\mathbf{X}] = \frac{1}{2}\int_{-T}^{T^\star} \left\| \partial_t \mathbf{X} + \mathcal{T}[\mathbf{X}] + \mathcal{A}\frac{\delta\mathcal{H}}{\delta\mathbf{X}} \right\|^2_{\mathcal{A}^+} \mathrm{d}t\,, \tag{S21}$$

and it can be written as

$$\begin{aligned} S^1_T[\mathbf{X}] \;&=\; \frac{1}{2}\int_{-T}^{T^\star} \left\| \partial_t \mathbf{X} + \mathcal{T}[\mathbf{X}] - \mathcal{A}^\dagger\frac{\delta\mathcal{H}}{\delta\mathbf{X}} \right\|^2_{\mathcal{A}^+} \mathrm{d}t \\ &+\; \int_{-T}^{T^\star} \left(\partial_t \mathbf{X} + \mathcal{T}[\mathbf{X}]\,, \mathcal{A}^+\mathcal{A}\,\frac{\delta\mathcal{H}}{\delta\mathbf{X}} \right) \mathrm{d}t \;+ \\ &+\; \int_{-T}^{T^\star} \left(\frac{\delta\mathcal{H}}{\delta\mathbf{X}}[\mathbf{X}], \mathcal{A}^+\mathcal{A}\,\left(\partial_t \mathbf{X} + \mathcal{T}[\mathbf{X}]\right) \right) \mathrm{d}t \;= \\ &=\; \frac{1}{2}\int_{-T}^{T^\star} \left\| \partial_t \mathbf{X} + \mathcal{T}[\mathbf{X}] - \mathcal{A}^\dagger\frac{\delta\mathcal{H}}{\delta\mathbf{X}} \right\|^2_{\mathcal{A}^+} \mathrm{d}t \;+ \\ &+\; 2\int_{-T}^{T^\star} \left(\frac{\delta\mathcal{H}}{\delta\mathbf{X}}[\mathbf{X}], \mathcal{A}^+\mathcal{A}\mathcal{A}\frac{\delta\mathcal{H}}{\delta\mathbf{X}} \right) \mathrm{d}t\,. \end{aligned} \tag{S22}$$

By considering that on the trajectory which leads the first integral to zero, we have that $(\partial_t \mathbf{X} + \mathcal{T}[\mathbf{X}] = \mathcal{A}^\dagger \delta\mathcal{H}/\delta\mathbf{X})$ which guarantees

$$\frac{\mathrm{d}\mathcal{H}}{\mathrm{d}t} = \left(\frac{\delta\mathcal{H}}{\delta\mathbf{X}}, \mathcal{A}\frac{\delta\mathcal{H}}{\delta\mathbf{X}} \right)\,, \tag{S23}$$

Therefore one has

$$S^1_T[\mathbf{X}] = \frac{1}{2}\int_{-T}^{T^\star} \left(\left\| \partial_t \mathbf{X} + \mathcal{T}[\phi] - \mathcal{A}^\dagger\frac{\delta\mathcal{H}}{\delta\mathbf{X}}[\mathbf{X}] \right\|^2_{\mathcal{A}^+} + 2\frac{\mathrm{d}\mathcal{H}}{\mathrm{d}t} \right) \mathrm{d}t\,. \tag{S24}$$

It is worth stressing that the action in Eq. (S21) can vanish only along deterministic paths, i.e. trajectories that the system can follow even in the absence of thermal fluctuations. By contrast, the nucleation branch corresponds to a fluctuation-driven path that enables the system to escape from the metastable basin. Along this branch, the action

cannot vanish, since without noise the formation of a critical bubble would be impossible. For this reason, the trajectory minimising the action along the nucleation branch only makes the first integral in Eq. (S22) vanish. This result aligns with the general theoretical framework developed in [8], which combines a Hamiltonian formalism with a large deviation theory (LDT) perspective on time reversibility. In particular, it is consistent with the prediction that energy increases monotonically along instanton trajectories, reflecting the characteristic structure of fluctuation paths.

Supplementary References

1. Wagner, W. & Pruß, A. The IAPWS Formulation 1995 for the Thermodynamic Properties of Ordinary Water Substance for General and Scientific Use. *Journal of Physical and Chemical Reference Data* **31,** 387–535 (2002).
2. Kunz, O. & Wagner, W. The GERG-2008 Wide-Range Equation of State for Natural Gases and Other Mixtures: An Expansion of GERG-2004. *Journal of Chemical & Engineering Data* **57,** 3032–3091 (2012).
3. Wilhelmsen, Ø. *et al.* Thermodynamic modeling with equations of state: present challenges with established methods. *Industrial & Engineering Chemistry Research* **56,** 3503–3515 (2017).
4. Magaletti, F., Gallo, M. & Casciola, C. M. Water cavitation from ambient to high temperatures. *Scientific reports* **11,** 20801 (2021).
5. Mulero, A., Cachadina, I. & Parra, M. Recommended correlations for the surface tension of common fluids. *Journal of Physical and Chemical Reference Data* **41,** 043105 (2012).
6. Lemmon, E. W., Huber, M. L. & McLinden, M. O. *REFPROP: Reference Fluid Thermodynamic and Transport Properties* NIST Standard Reference Database 23, Version 9.0. National Institute of Standards and Technology (Gaithersburg, MD, 2010).
7. Langer, J. & Turski, L. Hydrodynamic model of the condensation of a vapor near its critical point. *Physical Review A* **8,** 3230 (1973).
8. Bouchet, F. Is the Boltzmann equation reversible? A large deviation perspective on the irreversibility paradox. *Journal of Statistical Physics* **181,** 515–550 (2020).
