# Supplementary material for: Hydrodynamical pathways in the phase change of real fluids
Source: Commun Phys. 2026 Apr 27;9(1):220. doi: 10.1038/s42005-026-02639-y (PMC13323055; doi:10.1038/s42005-026-02639-y)
Supplement: Supplementary file 1 — Transparent Peer Review file [file 42005_2026_2639_MOESM1_ESM.pdf]

nature portfolio

# Peer Review File

# Hydrodynamical pathways in the phase change of real fluids.

Corresponding Author: Dr Mirko Gallo

**This file contains all reviewer reports in order by version, followed by all author rebuttals in order by version.**

Version 0:

Reviewer comments:

Reviewer #1

(Remarks to the Author)
The work by Gallo et al. combines large deviation theory, equilibrium thermodynamics, and fluctuating hydrodynamics to study nucleation in superheated fluids using the most probable nucleation path in phase space that connects metastable and stable phases. The structure of the system along the path is then characterized with the density and the velocity fields as well as with bulk and generalized pressure profiles. The results highlight the important of non-equilibrium (inertial) effects during nucleation, in contrast to classical nucleation theory that often neglects dynamical aspects.

Overall the paper is well written. The section "Theoretical Aspects" is highly technical and difficult to read for non-specialists, specially the sections "Transverse Decomposition of CLLNS" and and "Most likely Path of Nucleation". On the other hand, the introduction and the results are clearly written and are easy to follow. The paper is detailed enough as to make it possible for other researchers to reproduce the results. The agreement between theory and experiments for the boiling curves of water, helium, and nitrogen is remarkable and it helps to support the theoretical framework. The approach presented by Gallo et al. is new, it can in principle be extended to other systems and phase transitions, and it accurately predicts the boiling temperature in real fluids. I recommend publication in Communication Physics and have only a couple of comments for the authors.

- In my opinion, the presentation of the results would improve significantly if Fig. 3 is presented before Fig. 2 and Fig. 6 before Fig.5. It is much easier to understand the meaning of the profiles shown in Figs. 2 and 5 after having visualized the two-dimensional snapshots of the density and velocity profiles.

- If I understood it correctly, equations (1) and (2) play an important role since they determine the time evolution of the density and velocity fields. Equation (2) constitutes an approximation to the real dynamical evolution of the one-body fields. The effects of the interparticle interactions on the dynamics of the system are essentially encoded in the second and the third terms of the right hand side of Eq. (2). The third term, which is explicitly written in Eq. (3), is an equilibrium (adiabatic-like) force density field given by the of a free energy. Deviations from this equilibrium-like (adiabatic approximation) force density field due to the interparticle interactions are incorporated via shear and bulk viscous contributions, Eq. (5). There are higher order contributions (not considered here) that go beyond the adiabatic approximation and that can play a role in the nucleation process, specially at times and in regions for which the magnitude of the velocity profile is large. I think the paper should state it more clearly that Eq. (2) is an approximation to the real dynamics, and that it is primarily based on the assumption that the force density field originated from the interparticle interactions captures only small deviations from an equilibrium force field via Eq. (5).

- The meaning of the inset of Fig. 3c is only briefly explained in the main text but no mention is done in the caption of the figure. Also, I am not sure that I understand this inset. What is the meaning of each orange circle? Does each orange circle correspond to a simulation at a given time? If yes, would it be possible to color the circles according to the time?

- I think that a few quantities are undefined or at least they are not defined the first time that they appear. See for example, rho and v in Eq. (1); F, \cal D, and V in Eq. (2); k_B, I, and \nabla_X in Eq. (6); \Kappa in Eq. (7); \tilde\mu in Eq. (31).

- Figs. 3 and 6: What is the meaning of the arrow's color? Is there a color bar missing?

- What are the units of x and y in Figs. 3 and 6?

------------------
Other minor points
------------------

- What is the definition of $?_met$ quantities in Fig. 2?

- Eq.s => Eqs. in Pgs. 2 and 3.

- There are several minor formatting issues that the authors might want to correct. Examples include inconsistent use of "Eq. (?)" vs "Eq. ?", references with and without links to the actual papers in both the main text and the supplemental material, use of both full journal titles and abbreviated ones in the bibliography, inconsistent capitalization of journal names (e.g. Physical review letters and Physical Review Letters), inconsistent use of font types in units across the main text and the figures (just one example, see "[ns]" Fig. 1a vs "[\it{ns}]" Fig. 1b).

Reviewer #2

(Remarks to the Author)
The paper titled “Hydrodynamical pathways in the phase change of real fluids” by Mirko Gallo et al. presents a study of nucleation pathways in superheated fluids. The authors determine the most likely transition path from the metastable liquid to the stable vapour state based on the Capillary Landau-Lifshitz-Navier-Stokes Equations and the Large Deviation theory. The developed model extends previous approaches by including inertial and kinetic effects which is crucial to describe the collective nature of the compression wave as seems to be highly relevant for the nucleation process. Additionally to the theoretical model and its application within numerical simulations the manuscript also compared the predicted boiling temperatures with experimental data of Water, Nitrogen and Helium.

Overall, the presentation of the manuscript is clear and the derivations appear sound. I particularly liked that the (various) used concepts and theories are briefly introduced before guiding the reading to more detailed literature. To improve the already very good manuscript, I would encourage the authors to address the following minor points in a minor revision.

1) The manuscript shows that the developed theory and the correspondingly predicted boiling curves for Water, Helium and Nitrogen have excellent agreement with the experimental data. I wonder what the restrictions and limitations of the method are in practice. Is it the computational power or other approximations like the limit of small (compared to what?) epsilon to apply the Freidlin-Wentzell theory in Eq. (17). Could the authors please comment on that.

2) In my opinion it is interesting that the most likely transition path predicts the long-range acoustic waves during the nucleation process. Do the authors have any idea if this prediction could be verified experimentally? I assume it might be hard to resolve the necessary length and time scales.

3) I did not really get the reasoning in appendix A. What is the reason to consider the first integral in Eq. (49) to be zero and why would this reasoning only apply to Eq. (49) but not Eq. (48)? Could you please extend the description of this detail.

4) Fig.3 and 6: It would be helpful to also include the colour bar for the velocities.
Fig. 5(b): Do I understand it correctly that the different curves in the insert correspond to different times? A short comment in the caption would be useful.

5) After Eq. (4), “f_b(ρ, θ) the bulk (Helmholtz) free energy”: I think that the variable f is the corresponding energy density. Also I think the (Helmholtz) free energy F is never formally introduced.
Eq. (7): Please introduce \mathcal{K} as the kinetic energy.

6) Beginning of page 4, “As shown in [32, 37], the dominant term of P [X] (O(ε−1)) (see Eq. 17), with the subsequent terms (related to the Jacobian of the trasformation ξ = ξ[X]) scaling as O(ε0).”: The word transformation has a typo (missing letter n) and the sentence misses a verb.

Version 1:

Reviewer comments:

Reviewer #1

(Remarks to the Author)
The authors have responded satisfactorily to all points raised by the referees. In my opinion they have improved the manuscript and I am happy to recommend its publication in Communication Physics.

Reviewer #2

(Remarks to the Author)
I thank the authors for the thorough and detailed revision, which has addressed all my points. My recommendation is to publish this manuscript in its present form.

Minor point:
After eq. (4): "f_b(ρ, θ) its bulk density" should be rather called bulk free-energy density.

**Open Access** This Peer Review File is licensed under a Creative Commons Attribution 4.0 International License, which permits use, sharing, adaptation, distribution and reproduction in any medium or format, as long as you give appropriate credit to the original author(s) and the source, provide a link to the Creative Commons license, and indicate if changes were made.
In cases where reviewers are anonymous, credit should be given to 'Anonymous Referee' and the source.
The images or other third party material in this Peer Review File are included in the article's Creative Commons license, unless indicated otherwise in a credit line to the material. If material is not included in the article's Creative Commons license and your intended use is not permitted by statutory regulation or exceeds the permitted use, you will need to obtain permission directly from the copyright holder.
To view a copy of this license, visit https://creativecommons.org/licenses/by/4.0/

**Replay Referee # 2**

**COMMSPHYS-25-1916-T**

**Hydrodynamical pathways in the phase change of real fluids.**

Gallo, Mirko; Occhioni, Filippo ; Daniele, Riccardo; Casciola, Carlo Massimo

*Comments from the Referee:*

*The paper titled "Hydrodynamical pathways in the phase change of real fluids" by Mirko Gallo et al. presents a study of nucleation pathways in superheated fluids. The authors determine the most likely transition path from the metastable liquid to the stable vapour state based on the Capillary Landau-Lifshitz-Navier-Stokes Equations and the Large Deviation theory. The developed model extends previous approaches by including inertial and kinetic effects which is crucial to describe the collective nature of the compression wave as seems to be highly relevant for the nucleation process. Additionally to the theoretical model and its application within numerical simulations the manuscript also compared the predicted boiling temperatures with experimental data of Water, Nitrogen and Helium.*

*Overall, the presentation of the manuscript is clear and the derivations appear sound. I particularly liked that the (various) used concepts and theories are briefly introduced before guiding the reading to more detailed literature. To improve the already very good manuscript, I would encourage the authors to address the following minor points in a minor revision.*

We thank the Referee for the time and care devoted to reviewing our manuscript, and in particular for the positive assessment of the clarity of the presentation and of the way the various concepts and theories are introduced before directing the reader to more detailed literature. We have carefully addressed all the minor points raised, and all revisions have been implemented in the new version of the manuscript, with changes highlighted in blue to facilitate reading.

1) *The manuscript shows that the developed theory and the correspondingly predicted boiling curves for Water, Helium and Nitrogen have excellent agreement with the experimental data. I wonder what the restrictions and limitations of the method are in practice. Is it the computational power or other approximations like the limit of small (compared to what?) epsilon to apply the Freidlin-Wentzell theory in Eq. (17). Could the authors please comment on that.*

We thank the referee for raising this point, which allows us to clarify an important aspect of the present approach. The computational cost of calculating the transition pathways is, in fact, very modest and can be handled on a standard laptop in less than one hour.
By contrast, as correctly pointed out by the referee, the small-$\epsilon$ approximation is more delicate, since in realistic systems the noise intensity may not be small. More generally, within Large Deviation Theory, it is notoriously difficult to obtain an *a priori* estimate of the effective noise intensity. In the present context, $\epsilon$ should be compared with the typical magnitude of the action, which in nucleation problems can reasonably be expected to scale with the height of the free-energy barrier. Since the path probability scales as $P[X] \sim \exp[-S[X]/\epsilon]$, in the small-$\epsilon$ regime, it is exponentially dominated by the

minimum of the action. For the cases considered here, this leads to a reasonable dimensionless estimate of the noise strength, $\epsilon^*$, with $\epsilon^* \sim k_B\theta/\Delta\Omega^\star \sim 10^{-2}$, with $\Delta\Omega^\star$ the free-energy barrier, fairly consistent with the small noise hypothesis.

Assessing the quantitative role of fluctuations beyond this asymptotic regime will be the subject of future work, where the nucleation pathways computed within our framework can help design effective rare-event sampling strategies, a perspective we intend to pursue in the near future.

A comment on this point is now reported in the new version of the manuscript.

*2) In my opinion it is interesting that the most likely transition path predicts the long-range acoustic waves during the nucleation process. Do the authors have any idea if this prediction could be verified experimentally? I assume it might be hard to resolve the necessary length and time scales.*

The referee is correct in noting that a direct experimental verification of these predictions would be challenging, due to the very small spatial and temporal scales involved. We are not experimentalists, and at present we cannot assess whether such measurements are technically feasible. The predicted dynamics involves compressive waves with frequencies in the GHz range, decay times on the order of nanoseconds, and characteristic wavelengths of a few hundred nanometers, which are difficult to resolve experimentally.

One may nonetheless speculate about experiments in which a metastable fluid is externally excited by waves with characteristics similar to those predicted by the transition pathways, in order to probe whether such perturbations have a measurable impact on the liquid–vapor phase transition, for instance through changes in the nucleation rate or in the effective boiling temperature.

We hope that the present work can help motivate future experimental efforts aimed at exploring these possibilities.

On the theoretical and numerical side, recent work has provided further support to the present framework. In particular, the nonclassical C-shaped nucleation pathways predicted by this theory have been observed in atomistic simulations of droplet nucleation for LJ prototype fluids (*Wu, Yijian, et al., "Nonclassical Nucleation Pathways in Liquid Condensation Revealed by Simulation and Theory," Physical Review Letters 2026*), in qualitative agreement with our predictions. Direct and quantitative comparisons with molecular dynamics simulations represent a natural and important direction for future work.

A brief comment highlighting these perspectives has been added to the Conclusions.

*3) I did not really get the reasoning in appendix A. What is the reason to consider the first integral in Eq. (49) to be zero and why would this reasoning only apply to Eq. (49) but not Eq. (48)? Could you please extend the description of this detail.*

The key point lies in the fundamentally different nature of the trajectories described by Eqs.~(48) and (49). The action appearing in Eq.~(48) can vanish only along a purely deterministic path, namely a trajectory that the system can follow even in the absence of thermal fluctuations. Such paths correspond to standard Navier–Stokes–like solutions characterised by positive dissipation, and therefore do not require noise to be realised, yielding a zero contribution to the action. By contrast, the nucleation branch corresponds to a fluctuation-driven (uphill) trajectory that allows the system to escape from the metastable basin. Along this branch, the dynamics effectively requires a rare noise realisation that locally overcomes dissipation, which can be interpreted as an effective negative dissipation over the duration of the event. Consequently, the action along the nucleation branch cannot vanish: if it did, the trajectory could be realized without noise and nucleation would never occur. In other words, in the absence of thermal fluctuations, the formation of a critical bubble would be impossible. For this reason, the trajectory minimizing the action along the nucleation branch cannot make the integral in Eq.~(49) vanish, and the reasoning applicable to Eq.~(48) does not extend to Eq.~(49).

We have clarified this point and expanded the corresponding discussion in Appendix A in the revised version of the manuscript.

*4) Fig.3 and 6: It would be helpful to also include the colour bar for the velocities.*
*Fig. 5(b): Do I understand it correctly that the different curves in the insert correspond to different times? A short comment in the caption would be useful.*

Thank you for these suggestions. For Figs. 3 and 6, we agree that including a colour bar for the velocity magnitude improves the readability of the figures, and we have now added it.
Regarding Fig. 5(b), yes, the different curves in the inset correspond to different times. The inset is meant to illustrate the behaviour at long times and over increasingly large spatial scales, in order to highlight the approach to the asymptotic Rayleigh–Plesset limit. We have now clarified this point explicitly in the figure caption and improved the explanation in the text accordingly.

*5) After Eq. (4), "f_b(ρ, θ) the bulk (Helmholtz) free energy": I think that the variable f is the corresponding energy density. Also I think the (Helmholtz) free energy F is never formally introduced. Eq. (7): Please introduce \mathcal{K} as the kinetic energy.*

We thank the Referee for these remarks. We have clarified that $f_b(\rho, \theta)$ denotes the bulk (Helmholtz) free-energy density, and we have now explicitly introduced the corresponding (Helmholtz) free energy $F[\rho, \theta]$ in the revised manuscript. In addition, in Eq. (7) we now explicitly introduce $\mathscr{K}[\rho, \mathbf{v}]$ as the kinetic energy.

*6) Beginning of page 4, "As shown in [32, 37], the dominant term of P [X] (O(ε−1)) (see Eq. 17), with the subsequent terms (related to the Jacobian of the trasformation ξ = ξ[X]) scaling as O(ε0).": The word transformation has a typo (missing letter n) and the sentence misses a verb.*

We thank the Referee for pointing this out. The typo has been corrected, and the sentence has been revised to include the missing verb in the revised version of the manuscript.

We thank the Referee for the time and effort devoted to reviewing our manuscript and for the constructive comments provided. We hope that the revised version addresses all the points raised and that the manuscript is now suitable for publication in *Communications Physics*.

**Replay Referee # 1**

**COMMSPHYS-25-1916-T**

**Hydrodynamical pathways in the phase change of real fluids.**

Gallo, Mirko; Occhioni, Filippo ; Daniele, Riccardo; Casciola, Carlo Massimo

*Comments from the Referee:*

*The work by Gallo et al. combines large deviation theory, equilibrium thermodynamics, and fluctuating hydrodynamics to study nucleation in superheated fluids using the most probable nucleation path in phase space that connects metastable and stable phases. The structure of the system along the path is then characterized with the density and the velocity fields as well as with bulk and generalized pressure profiles. The results highlight the important of non-equilibrium (inertial) effects during nucleation, in contrast to classical nucleation theory that often neglects dynamical aspects.*

*Overall the paper is well written. The section "Theoretical Aspects" is highly technical and difficult to read for non-specialists, specially the sections "Transverse Decomposition of CLLNS" and and "Most likely Path of Nucleation". On the other hand, the introduction and the results are clearly written and are easy to follow. The paper is detailed enough as to make it possible for other researchers to reproduce the results. The agreement between theory and experiments for the boiling curves of water, helium, and nitrogen is remarkable and it helps to support the theoretical framework. The approach presented by Gallo et al. is new, it can in principle be extended to other systems and phase transitions, and it accurately predicts the boiling temperature in real fluids. I recommend publication in Communication Physics and have only a couple of comments for the authors.*

We thank the Referee for the time and effort devoted to reviewing our manuscript, and for recommending its publication. Below, we provide a point-by-point response to all comments raised, as well as to the insightful suggestions aimed at improving the readability and strengthening the overall message of the paper. All revisions have been implemented in the new version of the manuscript, and changes are highlighted in blue for ease of reference.

1) *In my opinion, the presentation of the results would improve significantly if Fig. 3 is presented before Fig. 2 and Fig. 6 before Fig.5. It is much easier to understand the meaning of the profiles shown in Figs. 2 and 5 after having visualized the two-dimensional snapshots of the density and velocity profiles.*

We have followed the Referee's suggestion, and in the revised version, the figures have been introduced in the recommended order.

2) *If I understood it correctly, equations (1) and (2) play an important role since they determine the time evolution of the density and velocity fields. Equation (2) constitutes an approximation to the real dynamical evolution of the one-body fields. The effects of the interparticle interactions on the dynamics of the system are essentially encoded in the second and the third terms of the right hand side of Eq. (2). The third term, which is explicitly written in Eq. (3), is an equilibrium (adiabatic-like) force density field given by the gradient of a free energy. Deviations from this equilibrium-like (adiabatic approximation)*

*force density field due to the interparticle interactions are incorporated via shear and bulk viscous contributions, Eq. (5). There are higher order contributions (not considered here) that go beyond the adiabatic approximation and that can play a role in the nucleation process, specially at times and in regions for which the magnitude of the velocity profile is large. I think the paper should state it more clearly that Eq. (2) is an approximation to the real dynamics, and that it is primarily based on the assumption that the force density field originated from the interparticle interactions captures only small deviations from an equilibrium force field via Eq. (5).*

We agree with the referee's interpretation. Equation (2) represents an approximation to the microscopic dynamics. In the present formulation, interparticle interactions are described through an equilibrium force density based on squared-gradient theory, which assumes that the local thermodynamic state can be characterised by equilibrium relations evaluated at the instantaneous values of the hydrodynamic fields (local equilibrium approximation). The squared-gradient description is expected to be robust in the present case, since the interfacial thickness is much larger than the typical decay length of the interaction potentials (see, for example, Español, Pep, *J. Chem. Phys.*2001). The Newtonian dissipative contribution further assumes small departures from local equilibrium, leading to a linear constitutive relation between the viscous stress tensor and the velocity gradients. Higher-order non-equilibrium corrections to the thermodynamic fluxes, corresponding to departures from local equilibrium, are neglected here.

Thanking the referee for this interesting comment, we have added a brief comment highlighting these limitations to the Conclusions.

3) *The meaning of the inset of Fig. 3c is only briefly explained in the main text but no mention is done in the caption of the figure. Also, I am not sure that I understand this inset. What is the meaning of each orange circle? Does each orange circle correspond to a simulation at a given time? If yes, would it be possible to color the circles according to the time?*

We think the referee was referring to Fig. 1c. As correctly pointed out, the orange circles shown in the inset of Fig. 1c correspond to individual realisations of the stochastic nucleation path obtained from fluctuating hydrodynamics simulations (CLLNS). Each circle represents the system state at a given time. Following the referee's suggestion, in the revised version of the manuscript, we have updated the figure by colouring the circles according to time, and we have added a clarifying comment in the main text together with an updated figure caption to better explain the meaning of the inset.

4) *I think that a few quantities are undefined or at least they are not defined the first time that they appear. See for example, rho and v in Eq. (1); F, \cal D, and V in Eq. (2); k_B, I, and \nabla_X in Eq. (6); \Kappa in Eq. (7); \tilde\mu in Eq. (31).*

We thank the Referee for bringing this to our attention. All the quantities mentioned have now been properly defined in the revised manuscript.

5) *Figs. 3 and 6: What is the meaning of the arrow's color? Is there a color bar missing?*

We thank the Referee for pointing this out. The arrow color represents the velocity magnitude. The figure has now been modified accordingly, and the corresponding color bar has been added in the revised version.

6) *What are the units of x and y in Figs. 3 and 6?*

The units are nanometers and are now explicitly stated in the revised manuscript and specified on the new figures.

**Minor points**

*1) What is the definition of $?_met$ quantities in Fig. 2?*

The subscript “met” refers to the metastable fields, corresponding to the liquid state. We have amended the figure coherently with the definition in the text and explained this point.

*2) Eq.s => Eqs. in Pgs. 2 and 3.*

Thank you for pointing this out. The typo has been corrected, and “Eq.s” has been replaced with “Eqs.” on pages 2 and 3 in the revised version of the manuscript.

3) *There are several minor formatting issues that the authors might want to correct. Examples include inconsistent use of "Eq. (?)" vs "Eq. ?", references with and without links to the actual papers in both the main text and the supplemental material, use of both full journal titles and abbreviated ones in the bibliography, inconsistent capitalization of journal names (e.g. Physical review letters and Physical Review Letters), inconsistent use of font types in units across the main text and the figures (just one example, see "[ns]" Fig. 1a vs "[\it{ns}]" Fig. 1b).*

We thank the Referee for pointing out these issues. All the minor formatting inconsistencies mentioned have been carefully corrected in the revised version of the manuscript, both in the main text and in the Supplemental Material.

We thank the Referee for the time and effort devoted to reviewing our manuscript and for the constructive comments provided. We hope that the revised version addresses all the points raised and that the manuscript is now suitable for publication in *Communications Physics*.
